# Revisiting the Evolution and Taxonomy of *Clostridia*, a Phylogenomic Update

**DOI:** 10.1093/gbe/evz096

**Published:** 2019-05-10

**Authors:** Pablo Cruz-Morales, Camila A Orellana, George Moutafis, Glenn Moonen, Gonzalo Rincon, Lars K Nielsen, Esteban Marcellin

**Affiliations:** 1 Australian Institute for Bioengineering and Nanotechnology, The University of Queensland, St Lucia, Australia; 2 Zoetis, Parkville, Victoria, Australia; 3 Joint BioEnergy Institute, Emeryville, CA

**Keywords:** *Clostridium*, phylogenomics, pangenome, taxonomy

## Abstract

*Clostridium* is a large genus of obligate anaerobes belonging to the *Firmicutes* phylum of bacteria, most of which have a Gram-positive cell wall structure. The genus includes significant human and animal pathogens, causative of potentially deadly diseases such as tetanus and botulism. Despite their relevance and many studies suggesting that they are not a monophyletic group, the taxonomy of the group has largely been neglected. Currently, species belonging to the genus are placed in the unnatural order defined as *Clostridiales*, which includes the class *Clostridia*. Here, we used genomic data from 779 strains to study the taxonomy and evolution of the group. This analysis allowed us to 1) confirm that the group is composed of more than one genus, 2) detect major differences between pathogens classified as a single species within the group of authentic *Clostridium* spp. (sensu stricto), 3) identify inconsistencies between taxonomy and toxin evolution that reflect on the pervasive misclassification of strains, and 4) identify differential traits within central metabolism of members of what has been defined earlier and confirmed by us as cluster I. Our analysis shows that the current taxonomic classification of *Clostridium* species hinders the prediction of functions and traits, suggests a new classification for this fascinating class of bacteria, and highlights the importance of phylogenomics for taxonomic studies.

## Introduction

*Clostridia* are an important genus of Gram-positive, often anaerobic, rod shaped, spore-forming bacteria. The group includes important human and animal pathogens such as *C. botulinum*, *C. tetani*, *and C. difficile* as well as industrially relevant microorganisms such as *C. acetobutylicum*. The importance of the genus is reflected by the more than 42,000 entries in the PubMed database, and about 1,700 genome sequences from this group deposited in the GenBank database.

Early molecular analyses in the 1970s demonstrated considerable diversity and ambiguities among the genus (Johnson and Francis 1975). In fact, this early classification of the genus *Clostridium* does not respect the identity thresholds established for 16s rRNA (Rossi-Tamisier et al. 2015), a widely used taxonomic marker. In consequence, this classification has been revisited several times (Collins et al. 1994; Yutin and Galperin 2013; Lawson 2016). Currently, it is well known that there are at least three *C. botulinum* lineages and that *C. difficile* belongs to a distantly related genus leading to the recent reclassification of *C. difficile* as a *Clostridioides difficile* (Lawson et al. 2016). Furthermore, the genus *Sarcina* (Skerman et al. 1980) has been phylogenetically located within the “cluster I” (sensu stricto) group, which is widely accepted as the “true” *Clostridium* genus. The paroxysm of the conflicting organization of the *Clostridium* genus is the fact that the *Sarcina* genus was proposed before (Goodsir 1842) the *Clostridium* genus (Prazmowski 1880), giving priority to the name *Sarcina* for the whole genus. Although such change may be excessive and could cause a great deal of confusion, it highlights the need to revisit the taxonomy using modern approaches (see Lawson and Rainey 2016; Tindall 2016 for an interesting discussion on this subject).

The recent availability of sequenced genomes provides a new opportunity to revisit the clostridial taxonomy beyond 16S rRNA-based classification sequencing. Such an opportunity enables a comprehensive taxonomic and evolutionary analysis to confirm that they are not a monophyletic group and there is a need to redefine the group taxonomically.

In this work, we have compiled the genomes classified as “*Clostridium*” and “*Clostridioides*” in the GenBank database (Benson et al. 2006) to identify a set of conserved genes that were used to define taxonomy. Once the classification was established, we focused on what has been called “cluster I” species (sensu stricto) (Lawson and Rainey (2016)) to identify differences between the core/pan genomes of cluster I strains and to reveal general evolutionary trends and specific traits linked to adaptation to different lifestyles.

## Materials and Methods

All genomes assemblies were downloaded from the NCBI FTP site and filtered by number of contigs (cut-off <=400), N50 (>=20,000 bases), and completeness (>=80%) based on benchmarking Universal Single-Copy Orthologs (Waterhouse et al. 2017) implemented in QUAST v5.0.2 (Gurevich et al. 2013) resulting in 779 genomes which were annotated in RAST (Aziz et al. 2008). The conserved proteins present in the selected genomes were identified using BPGA v1.3 (Chaudhari et al. 2016) with an identity cut-off of 0.4 for clustering of groups of orthologs using Usearch (Edgar 2010).

The resulting 27 groups of orthologs were aligned using Muscle v3.8 (Edgar 2004) and the alignments were manually curated and concatenated using SeaView v4 (Gouy et al. 2010). The final amino acid matrix included 12,836 amino acids. The best amino acid substitution model for each of the 27 partitions (supplementary table S1, Supplementary Material online) was selected using the ModelFinder tool implemented in IQ-tree (Kalyaanamoorthy et al. 2017) and the phylogeny was constructed using IQ-tree (Nguyen et al. 2015), using the partitioned models with 10,000 bootstrap replicates.

Pangenome analysis of cluster I subgroups was performed using BPGA following the same approach described above. Homologs of BotA and AroA were mined and retrieved from the database using BlastP (Altschul et al. 1990) with an *e*-value cut-off of 1E-9 and bit score of 200. Phylogenetic trees for clostridial toxins and AroA were obtained using the same approach. Synteny analysis was performed using CORASON-BGC (Cruz-Morales et al. 2017) with an *e*-value cut-off of 1E-9 and a bit score of 200.

The full noncollapsed aroA and species trees are available as supplementary Tree 1, Supplementary Material online, deposited at TreeBASE (Vos et al. 2012): http://purl.org/phylo/treebase/phylows/study/TB2:S23279. Last accessed May 13, 2019.

## Results and Discussion

### General Taxonomy

We first retrieved more than 1,700 genomes and draft genomes deposited as “*Clostridium*” and “*Clostridioides*” from the GenBank as of July 2017. The data set was filtered by removing low-quality genomes (genomes with more than 400 contigs) and by eliminating redundancy at the strain level. This filtering resulted in a subset of 779 genomes (supplementary table S1, Supplementary Material online) used hereafter. We used the taxonomic definition of clostridial “clusters” as reference and annotated those strains with a species name accordingly (Tamburini et al. 2001; Liou et al. 2005; Rainey et al. 2006; Sakuma et al. 2006; Warren et al. 2006; Slobodkina et al. 2008; Shiratori et al. 2009; Bowman et al. 2010; Jung et al. 2010).

**Fig. 1.**
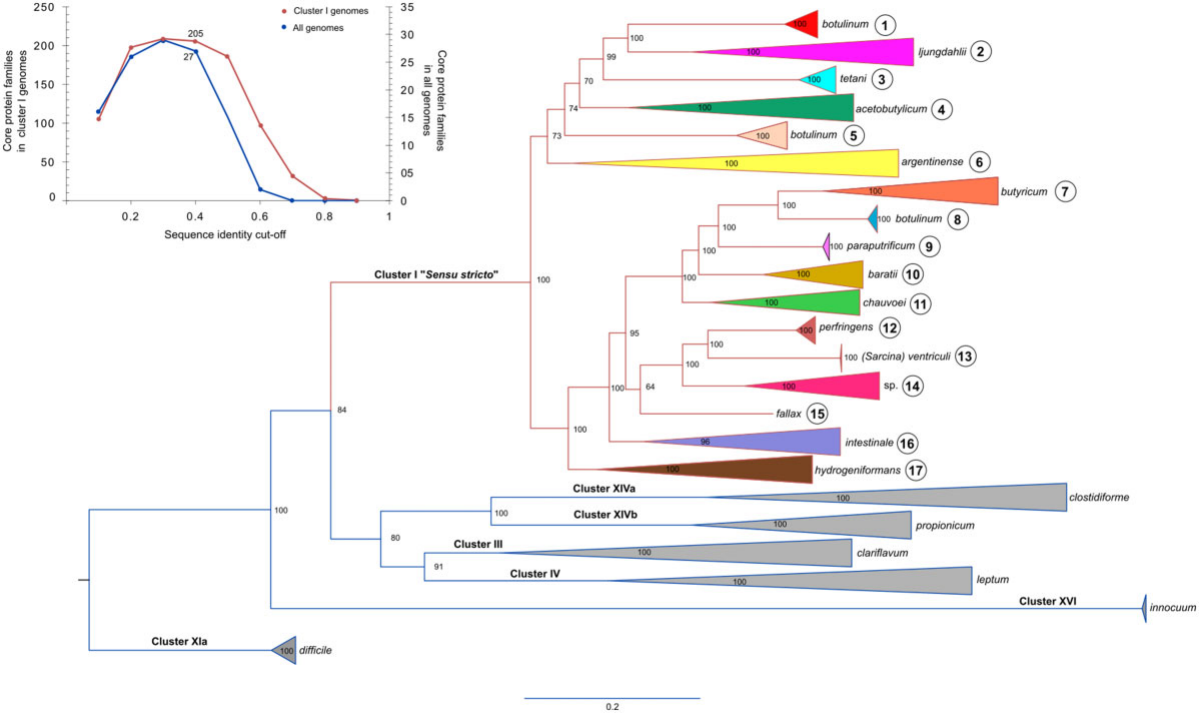
—Phylogenetic reconstruction of *Clostridium* species. Left corner: core protein families identified at different sequence identity cut-offs in all genomes and in genomes located in cluster I. The phylogeny was constructed using 27 markers conserved across 779 genomes (supplementary table S1, Supplementary Material online) deposited in the GenBank database and taxonomically defined as *Clostridium*. The main clades outside (blue lines) and within (369 taxa, red lines) the cluster I sensu stricto group (real clostridia) have been collapsed and defined as 17 taxonomic subgroups (table 1). Branch support is shown at each node. Uncollapsed clades for subgroups 1–17 are shown in supplementary figures S1–S14, Supplementary Material online.

From this subset of genomes, we calculated the core genome at different sequence identity cut-offs and selected the largest set of core proteins that could confidently be used for phylogenomic analysis (fig. 1) . Using this approach we identified 27 conserved protein sequences (supplementary table S2, Supplementary Material online) that were used for the construction of a clostridial species tree (fig. 1). This tree defined seven major clades that were consistent with the previous established clostridial “clusters” classification (Rainey et al. 2006). Accordingly, clusters III, IV, Xia, XIVa, XIVb, and XVI were distantly related to cluster I (sensu stricto), which contains 369 strains including *C. butyricum*, the type strain for the genus, the most toxin-producer pathogens and industrially relevant strains, but clearly excludes *difficile* species. Based on this analysis, we defined the members of cluster I as the authentic members of the *Clostridium* genus.

Cluster I was further divided into 17 subgroups (table 1) using the species tree presented in figure 1. Our analysis also showed that strains named *C. botulinum* are found in subgroups 1, 5, and 8 (table 1). These clades include *C. botulinum* strains defined by their toxin types as A/B/F (subgroup 1), C/D/CD (subgroup 5), and E (subgroup 8). Clades 1 and 5 also include other species namely: *C. sporogenes* in clade 1 and *C. haemolyticum* and *C. novyi* in clade 5.

**Table 1:** Subgroup Core Genome Analysis

**Subgroup** ^a^	Number of Strains	Species	Proteins Families in Subgroup Core	**Core of Cores** ^b^		
				Proteins in Core	Accessory Proteins	**Unique Proteins** ^c^
1	106	*botulinum* A, B, F*, sporogenes*	890	205	673	2
2	21	*autoethanogenum*, *carboxidivorans*, *coskatii*, *drakei*, *kluyveri*, *ljungdahlii*, *magnum*, *ragsdalei*, *scatologenes*, *tyrobutyricum*	1,071	205	755	99
3	10	*tetani*	2,214	205	1,115	877
4	16	*acetobutylicum*, *akagii*, *arbusti*, *aurantibutyricum*, *felsineum*, *pasteurianum*, *roseum*	1,063	205	752	96
5	42	*botulinum* C and D, *haemolyticum*, *novyi*	1,367	205	824	324
6	11	*argentinense*, *collagenovorans*, *estertheticum*, *proteolyticum*, *senegalense*, *sulfidigenes*, *tepidiprofundi*, *tunisiense*	462	205	245	4
7	48	*beijerinckii*, ***butyricum****(Clostridium type strain)*, *puniceum*, *saccharobutylicum*, *saccharoperbutylacetonicum*	1,319	205	925	178
8	20	*botulinum* E	994	205	769	10
9	4	*paraputrificum*	2,704	205	1,452	1,021
10	8	*baratii*, *colicanis*	1,350	205	1,037	94
11	15	*chauvoei*, *disporicum*, *sartagoforme*, *saudiense*, *septicum*	1,028	205	779	32
12	55	*perfringens*	2,044	205	1,276	547
13	3	*(Sarcina) ventriculi*	2,040	205	1,091	732
14	2	Spp.	1,505	205	1,043	242
15	1	*fallax*	2,415	205	1,336	854
16	3	*cavendishii*, *intestinale*	1,500	205	1,104	178
17	5	*algidicarnis*, *cadaveris*, *hydrogeniformans*	992	205	719	58

a Subgroups within cluster I are defined by the species tree presented in figure 1.

b Calculated using representative proteins for each protein family in the core of the subgroups at an identity cut-off of 0.4.

c This category includes protein families that are only found in the core of a given subgroup, therefore, they represent subgroup-specific protein families.

Comparison of the overall synteny between *C. botulinum* strains from clades 1, 5, and 8 showed divergence among them. High synteny could be observed between *C. botulinum* strains from clade 1 and *C. sporogenes* as well as *C. botulinum* strains from clade 5 and *C. novyi*, respectively (supplementary fig. S15, Supplementary Material online). The fact that *C. sporogenes*, *C. novyi*, and *C. haemolyticum* species show little divergence with their respective *C. botulinum* relatives suggest that these strains are either artificially defined as distinct species or have just recently diverged. Together, these observations indicate that the strains defined as *C.botulinum* should be split into three species found within groups 1, 5, and 8. *Clostridiumbotulinum* strains in subgroups 1 and 5 may be called, *C. sporogenes* and *C. haemolyticum*, respectively, since these species have been previously defined, whereas strains within subgroup 8 may remain as members of the authentic *C. botulinum* species. However, as highlighted by Lawson (2016b), changing names of medically relevant organisms can cause great confusion in the healthcare community. As these three species produce *botulinum* neurotoxins, the change of name might be rejected under Rule 56a (5) of the International Code of Nomenclature of Prokaryotes (Parker et al. 2015), which states that “names whose application is likely to lead to accidents endangering health or life or both” can be rejected.

As this analysis uses draft genomes to include as many genomes as possible, and only 27 proteins were conserved among these genomes, the analysis was repeated using a smaller subset of high-quality genomes (179, N50 > 600 kb) to validate our results. As such, a higher number of conserved proteins (79) were obtained and used (supplementary table S3, Supplementary Material online). This new analysis (supplementary fig. S16, Supplementary Material online) showed that the taxonomic groups maintained the same distribution (tree topology) when using a data set of 179 or 779 genomes and a matrix containing 79 or 27 protein sequences respectively. The same clusters and cluster I subgroups were observed (supplementary figs. S16–S29, Supplementary Material online), with the exception of clades that disappeared as they did not pass the stringent genome quality cut-off (clusters IV and XVI, and subgroups 9, 13, 14, and 15 in cluster I).

In agreement with our species tree, a new calculation of the clostridial core genome using only the 369 genomes of cluster I strains (fig. 1) yielded a set of conserved protein families one order of magnitude larger than when using all the genomes in our database. Overall, our results demonstrate the large divergence of clusters III, IV, XIa, XIVa, XIVb, and XVI relative to cluster I and highlight the need for their reclassification into at least five new genera.

### Toxin Evolution

Pathogenic clostridia produce the highest number of life-threatening toxins of any genus. This includes enterotoxins that affect the gut, such as *C. difficile* toxins A and B, histotoxins that affect soft tissue such as *C. perfringens* and *C. septicum* alpha-toxins, and neurotoxins affecting nervous tissue such as tetanus (*C. tetani*) and botulinum (*C. botulinum*) toxins. Diseases range from gastroenteritis to abdominal disorders, colitis, muscle necrosis, soft tissue infections, tetanus, and botulism among others (Hatheway 1990). These toxin-encoding genes are often located on mobile genetic elements or in variable regions of the chromosome (Hatheway 1990; Petit et al. 1999; Skarin and Segerman 2011), resulting in gene transfer between species. Here, we analyzed different toxins evolution to compare taxonomy with phylogeny.

**Fig. 2.**
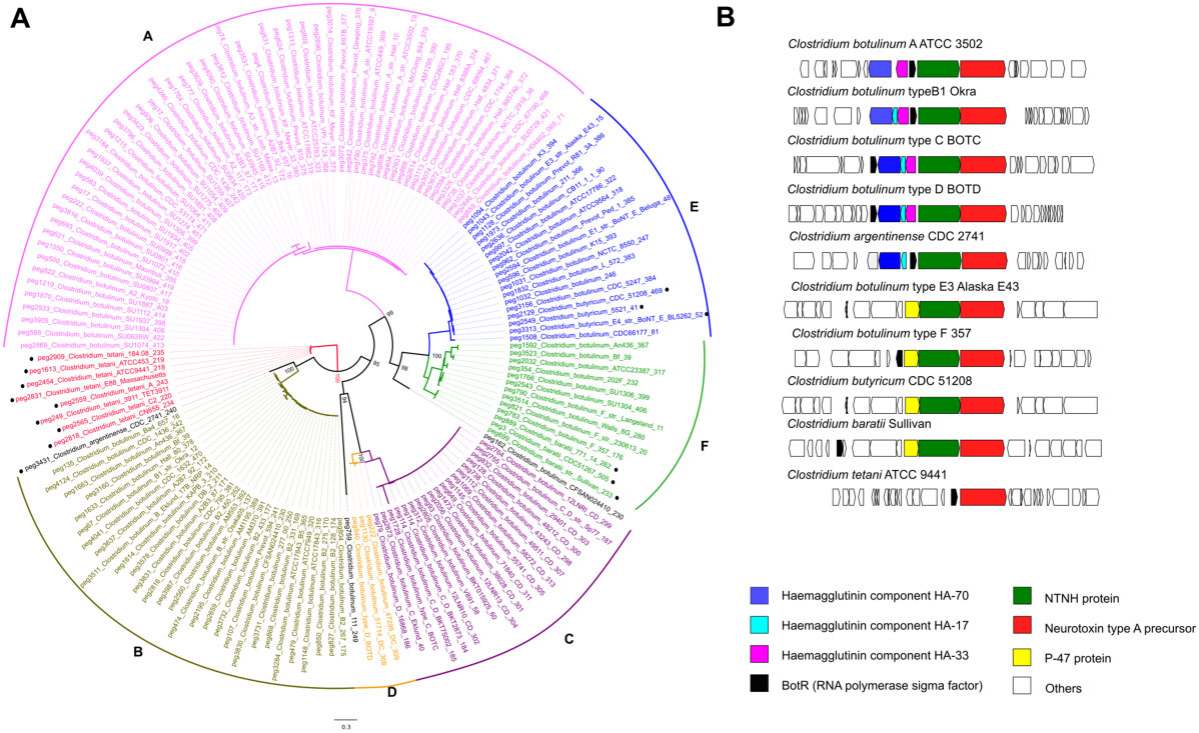
—(*A*) Phylogenetic reconstruction of BotA toxin proteins. Six A–F clades are consistent with previous reports. Including non-*botulinum* strains, *argentinense*, *tetani*, *butyricum*, and *baratti* (marked with a dot). Three new sequences (in black) account for new unclassified toxin diversity. (*B*) Genome context of BotA homologs found in cluster I strains.

The botulinum neurotoxin (BotA) for example, represents the most poisonous biological protein known and has been used as a phenotypic and genotypic marker for taxonomic classification. In fact, *C. botulinum* strains are often classified as members of groups A–F, in direct relationship with the production of antigenically distinguishable variants of the neurotoxin. In this work, homologs of BotA were found exclusively among members of cluster I and were distributed among *C. botulinum*, *C. tetani*, *C. argentinense*, *C. baratii*, and *C. butyricum* species. A phylogenetic reconstruction of these homologs (fig. 2*A*) showed little divergence except for three homologs: two on *botulinum* species and one on *C. argentinense* that seem to be more divergent.

The topology of the BotA phylogeny agrees with previous definitions of the *C. botulinum* subgroups A–F, with clades populated by strains with similar toxin types (i.e., clade A has only *C. botulinum* A strains, etc.). However, toxin markers were not consistent with the species tree, for which *C. botulinum* toxins types A, B, and F were in clade I while grouping independently in the toxin tree.

A gene context analysis (fig. 2*B*) showed two major synteny groups {A, B, C, D} and {E, F}. The presence of toxin accessory proteins (Lam et al. 2017) was found to be the main difference between them, namely hemagglutinin coding genes in groups A–D, and protein p47 in groups E and F. These observations are consistent with *C. botulinum* strains located in subgroups 1, 5, and 8 being distinct species that acquired the toxin genes by horizontal gene transfer hindering taxonomic classification.

The analysis of other important toxins also shows many horizontal gene transfer events of toxin genes between subgroups. *C. difficile* toxins A and B homologs were distributed among *C. difficile*, *C.sordellii*, *C. acetobutylicum*, and *C novyi* species (supplementary fig. S30, Supplementary Material online). TpeL from *C. perfringens* has been previously defined as a homolog of toxins A and B from *C. difficile* (Amimoto et al. 2007). However, TpeL proteins are largely divergent and therefore were not included in this analysis. Homologs of *C. perfringens* alpha-toxin were observed in *C. perfringens*, *C. novyi*, *C. botulinum* C and D, *C. baratii*, *C. hemolyticum*, *C. cavendishii*, *C. argentinense*, *C. sordellii*, and *C. dakarense* species (supplementary fig. S31, Supplementary Material online). Finally, *C. septicum* toxin alpha homologs were distributed among *C. septicum*, *C. novyi*, *C. haemolyticum*, and *C. botulinum* C and D species (supplementary fig. S32, Supplementary Material online). A summary of these findings can be found in supplementary table S4, Supplementary Material online. Interestingly, *C. botulinum* C and D (subgroup 5) also have *C. perfringens* and *C. septicum* alpha-toxins orthologs, whereas *C. botulinum* A, B, E, and F do not. According to these observations, we suggest that toxin production should not be used to define taxonomic groups, as it uncouples taxonomy from phylogeny.

### Core Genome Analysis

**Fig. 3.**
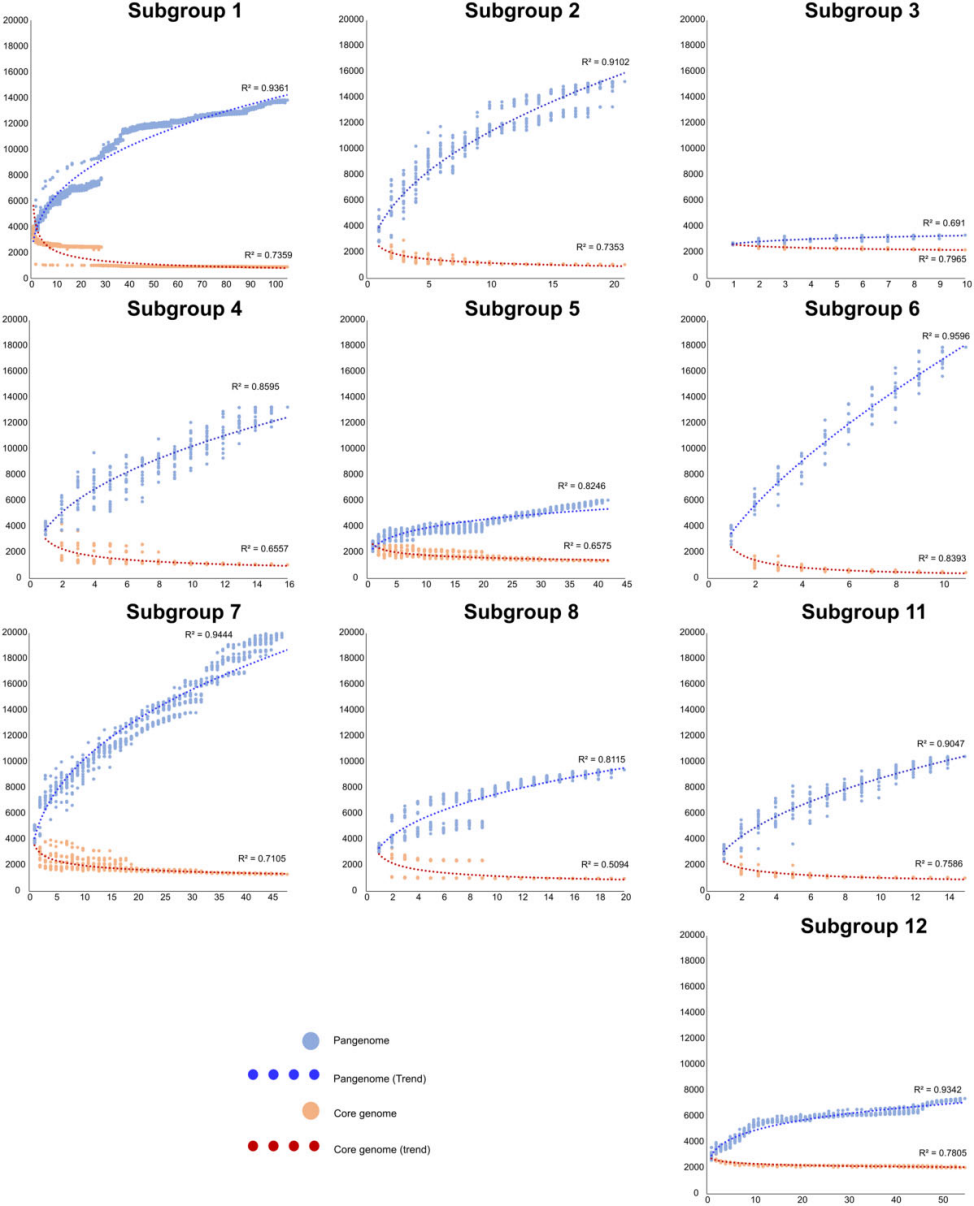
—Pangenome analysis of selected subgroups. The *y* axis shows the number of gene families and the *x* axis shows the number of genomes analyzed. The number of conserved genes was calculated by randomly adding genomes, with 20 replicates (if *n* > 20) or the same numbers as genomes (if *n* < 20). This analysis shows large differences in the genetic diversity of the subgroups, with less diversity and almost closed pangenomes in pathogenic subgroups.

Once the taxonomic framework was established, we used it to study the evolutionary dynamics and to identify general differences among the subgroups within cluster I. For this purpose, we calculated core/pangenomes for each subgroup having more than ten genomes (table 1). This analysis (fig. 3) showed that subgroups 3 (*C. tetani*), 5 (*C. botulinum* toxin group C and D, *C. haemolyticum*, and *C. novyi*), 8 (*C. botulinum* toxin group E), and 12 (*C. perfringens*) have almost closed pangenomes, implying loss of genetic diversity. This observation is consistent with the evolutionary dynamic observed in pathogenic species by other authors attributed to “Specialist” species (Georgiades and Raoult 2010).

In contrast, the remaining lineages showed open pangenomes. Subgroup 1, which includes important pathogens such as *C. botulinum* toxin groups A, B, and F, and the closely related *C. sporogenes* strains, has open pangenomes, implying larger genetic diversity and probably more recent adaptation to a pathogenic lifestyle. These observations further emphasize the presence of three distinct lineages among *C. botulinum* strains that may be reclassified as distinct species.

After establishing general differences between the evolutionary dynamics of the subgroups, we took a closer look at differences at the functional level. For this purpose, we extracted amino acid sequences of the core genes of each subgroup and identified conserved functions among them (i.e., a core of cores) and functions that are distinctive of each subgroup (table 1). This analysis revealed that cluster I has a core of 205 genes. As expected, many of these conserved genes are associated with housekeeping functions such as nucleotide biosynthesis, replication and repair (supplementary fig. S33, Supplementary Material online). Unique genes were abundantly classified as members of carbohydrate metabolism and for membrane transport. Interestingly, the largest number of accessory functions was related to amino acid metabolism, implying that multiple genes for this category are conserved at the subgroup level only. This observation is illustrated by the example described in the following section.

### The Divergence of the Shikimate Pathway in Pathogenic Clostridia

To investigate adaptive traits that could define differences within each subgroup, we mined the pangenomes for functions that were uniquely found in each group. From a taxonomic point of view, unique genomic traits are important as they can be used for the development of genetic markers and to identify distinctive phenotypes that can be used for classification. The rationale for searching unique traits within subgroups was that the use of such a large genomic database would enable, for the first time, to find unique functions conserved in all members of a subgroup but absent in other subgroups, thereby enabling to dissect for subgroup-specific adaptations. This was the case for the essential enzyme 3-phosphoshikimate 1-carboxyvinyltransferase (AroA), which was found in the pangenome of cluster I. AroA is part of the shikimate pathway and is essential for the biosynthesis of aromatic amino acids phenylalanine, tyrosine, and tryptophan. This seemed unusual given that all subgroup cores include AroA.

We reasoned that the presence of AroA among the pangenome may be due to 1) divergence among AroA orthologs beyond the cut-off for orthology defined in our pangenome strategy leading to fragmentation of the gene family or 2) duplication events in certain subgroups and divergence, which have been previously linked to adaptive evolution in bacteria (Schniete et al. 2018). To explore this idea, we searched for homologs of the AroA enzyme in all the strains from cluster I and found a single ortholog conserved in most strains. Thus, we assumed that AroA has divergently evolved within the cluster I species.

**Fig. 4.**
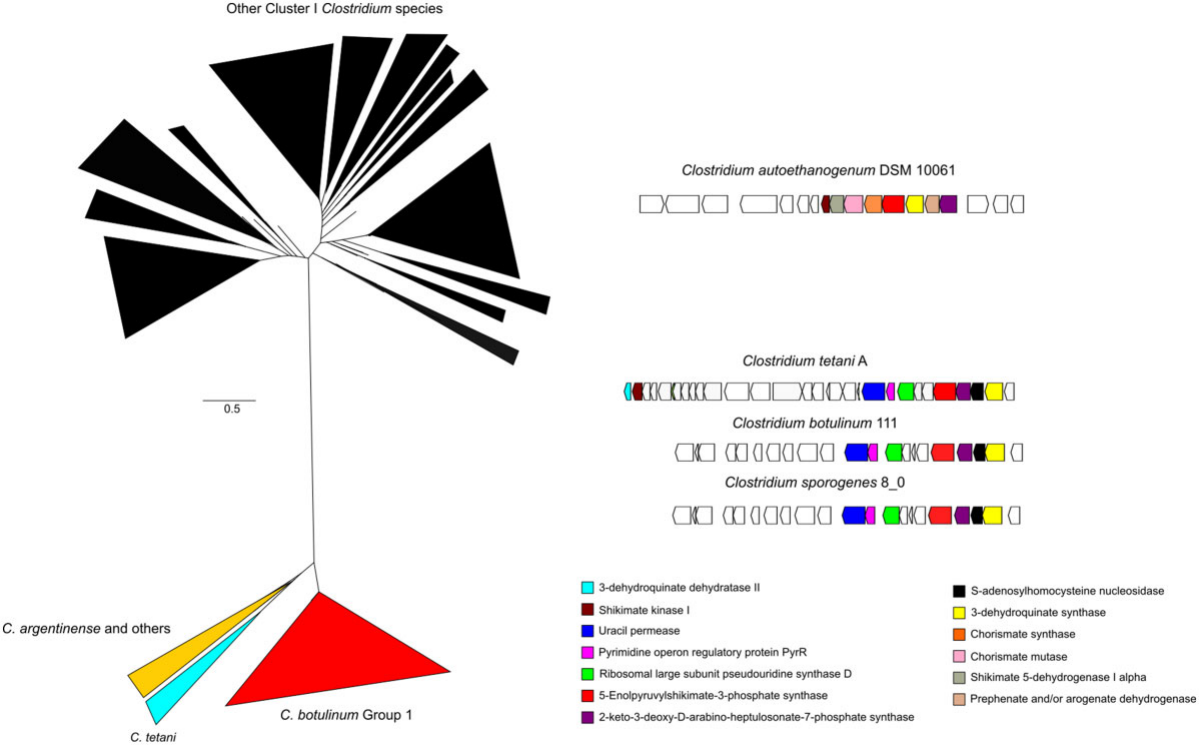
—Phylogenomic analysis of AroA in Clostridium group I. (*A*) The phylogeny shows that AroA, has significantly diverged in all members of the subgroup 3 (*C. tetani*; clear blue), subgroup 6 (*C. argentinense*; yellow) and subgroup 1 (*C. sporogenes*–*C. botulinum* B; red) from the rest of the subgroups in cluster I (black). The full tree is provided as supplementary Tree 2, Supplementary Material online. (*B*) Genome context of AroA homologs. *C. autoethanogenum* is shown as a typical Group I AroA genome context, whereas divergent homologs show a genome context that includes enzymes from pyrimidine metabolism.

Phylogenetic reconstruction of AroA (fig. 4*A*) confirmed the presence of two largely divergent AroA clades, one including homologs from strains in most subgroups and the other including subgroups 1, 3, and 11. Interestingly, most strains in this clade can colonize human hosts and are toxin-producing pathogens, except for *C. sporogenes*. Inspection of the genome context of representative AroA homologs from different subgroups (fig. 4*B*) revealed that despite sequence divergence, AroA homologs from *C. tetani*, *C. botulinum* toxin groups A, B and F, and *C. sporogenes* are located within a gene neighborhood that includes enzymes from the shikimate pathway. Thus, the gene context topology indicates that the function of these enzymes is linked to the production of aromatic amino acids. However, the divergent AroA homologs were found associated with the pyrimidine-associated regulator *pyrR* and a uracil permease. Such genomic organization suggests a link between aromatic amino acid biosynthesis and pyrimidine utilization. A recent in-depth molecular characterization of the *C. tetani* toxin production fermentation showed a potential link between extracellular uracil concentration and toxin production (Licona-Cassani et al. 2016). However, this link is yet to be fully understood.

Studies have also shown that *C. sporogenes*, a soil bacterium rarely pathogenic for humans (Inkster et al. 2011) although it may be found in the gut, and *C. botulinum* (cluster I), a toxin-producing pathogen, copiously produce tryptophan, phenylalanine and tyrosine. It has been suggested that secretion of these amino acids and intermediates of its degradation may influence intestinal permeability and systemic immunity of the host (Dodd et al. 2017). We speculate that the divergence in AroA may be related to the evolution of new metabolic interactions that do not affect the enzymatic activity of AroA, and unique regulation that occurs in clostridial species as a result of their ability to colonize hosts. Given the presence of this trait in pathogen and commensal strains, we reasoned that this trait likely evolved prior to the acquisition of toxin genes. Following the same argumentation, we suggest that *C. botulinum* subgroup 1 toxin groups A, B, and F have only recently evolved into pathogenic organisms.

By selecting amino acid biosynthesis to illustrate the use of the new classification, we show here that a correlation between traits, function gain and loss cannot be extracted from the current taxonomic classification of *Clostridium* species. Through this effort, we hope that our work serves to inspire the research community to study the evolution of clostridia at the genome-scale level and suggest a new classification for this fascinating class of bacteria.

## Conclusions

Here, we present an inclusive framework for phylogenomic analysis aimed at providing an updated view of the *Clostridium* genus. Our work shows that the current definition of clostridia encompasses a large and diverse group of species that is inconsistent with its definition as a genus. Instead, the group includes multiple genera. Furthermore, within the group I, arguably the authentic *Clostridium* genus, further taxonomic inconsistencies exist due to the use of BotA for taxonomic classification as a taxonomic marker. This has previously been observed by others (Yutin and Galperin 2013; Weigand et al. 2015; Lawson and Rainey 2016; Tindall 2016; Udaondo et al. 2017) but to the best of our knowledge, clostridial taxonomy and evolution has not been revisited using the opportunity offered by next-generation sequencing for phylogenomic reclassification until now. Given the pervasiveness of the misclassification in clostridial species, we wonder whether the current system of classification should be kept, or if it should be revisited and simplified using genomic analyses or a combination of “Classic” and phylogenomic approaches. Indeed, such synergy has already been shown to be useful in a smaller scale to reclassify former members of the genus *Clostridium* (Gerritsen et al. 2014).

The recent explosion of available annotated genomes offers an unprecedented opportunity to answer intriguing questions surrounding pathogenic clostridial evolution. For example, the incredible diversity and the number of toxins produced by some strains are yet to be fully understood. So is the astonishing potency of some of the toxins produced by these pathogens, which must confer an evolutionary advantage that remains to be elucidated.

## Supplementary Material

Supplementary data are available at *Genome Biology and Evolution* online.

## Supplementary Material

Supplementary_Matrial_evz096
