# Supplementary material for: Revisiting the Evolution and Taxonomy of Clostridia, a Phylogenomic Update
Source: Genome Biol Evol. 2019 May 10;11(7):2035–44. doi: 10.1093/gbe/evz096 (PMC6656338; doi:10.1093/gbe/evz096)
Supplement: Supplementary_Matrial_evz096 [file supplementary_matrial_evz096.docx]

Revisiting the evolution and taxonomy of Clostridia, a phylogenomic update.

(Supplementary material)

Pablo Cruz-Morales^1,a^, Camila A. Orellana^1^, George Moutafis^2^, Glenn Moonen^2^, Gonzalo Rincon^2^, Lars K Nielsen^1^ and Esteban Marcellin^1^

^1^Australian Institute for Bioengineering and Nanotechnology, The University of Queensland

^2^ Zoetis, 45 Poplar Rd, Parkville, Victoria Australia 3052

^a^ Present address: Joint BioEnergy Institute, 5885 Hollis St, Emeryville, CA 94608 USA

*Corresponding author: e.marcellin@uq.edu.au

**Table S1. Genomes included in the analysis**

| **Organism name** | **Completeness^1^** | **Clostridial cluster^2^** | **Contigs** | **Length** | **N50^3^** | **L50^4^** |
| --- | --- | --- | --- | --- | --- | --- |
| Clostridium_acetobutylicum_ATCC_824_2 | 98.03 | I | 2 | 4132880 | 3940880 | 1 |
| Clostridium_acetobutylicum_DSM_1731_61 | 98.03 | I | 3 | 4145581 | 3942462 | 1 |
| Clostridium_acetobutylicum_DSM_1732_527 | 98.43 | I | 55 | 4091215 | 270881 | 6 |
| Clostridium_acetobutylicum_EA_2018_57 | 98.03 | I | 2 | 4132226 | 3940230 | 1 |
| Clostridium_acetobutylicum_GXAS18_1_231 | 98.43 | I | 49 | 3796049 | 325351 | 4 |
| Clostridium_acetobutylicum_NCCB_24020_525 | 98.82 | I | 20 | 4098731 | 759218 | 2 |
| Clostridium_akagii_DSM_12554_164 | 97.64 | I | 50 | 4589511 | 266036 | 6 |
| Clostridium_algidicarnis_B3_167 | 98.03 | I | 1 | 3060291 | 3060291 | 1 |
| Clostridium_arbusti_SL206_70 | 96.46 | I | 243 | 3970278 | 31124 | 40 |
| Clostridium_argentinense_CDC_2741_240 | 98.43 | I | 20 | 4742562 | 418119 | 4 |
| Clostridium_aurantibutyricum_DSM_793_518 | 97.64 | I | 221 | 4922827 | 51056 | 31 |
| Clostridium_autoethanogenum_DSM_10061_356 | 97.64 | I | 1 | 4352446 | 4352446 | 1 |
| Clostridium_baratii_2789STDY5834907_337 | 97.64 | I | 50 | 3141705 | 1732372 | 1 |
| Clostridium_baratii_2789STDY5834956_336 | 98.43 | I | 30 | 3086202 | 440129 | 2 |
| Clostridium_baratii_771_14_262 | 98.43 | I | 39 | 3173174 | 211064 | 5 |
| Clostridium_baratii_CDC51267_505 | 98.43 | I | 2 | 3211717 | 3091050 | 1 |
| Clostridium_baratii_str._Sullivan_233 | 98.82 | I | 2 | 3338630 | 3153266 | 1 |
| Clostridium_baratii_XCM_325 | 98.43 | I | 28 | 3087740 | 857756 | 2 |
| Clostridium_beijerinckii_4J9_513 | 97.64 | I | 162 | 5888124 | 89920 | 20 |
| Clostridium_beijerinckii_ATCC_35702_SA_1_224 | 97.64 | I | 1 | 5999050 | 5999050 | 1 |
| Clostridium_beijerinckii_ATCC_39058_512 | 96.85 | I | 302 | 5953339 | 49706 | 37 |
| Clostridium_beijerinckii_BAS_B2_530 | 98.03 | I | 245 | 5982920 | 58724 | 32 |
| Clostridium_beijerinckii_BAS_B3_I_124_509 | 97.24 | I | 1 | 6123550 | 6123550 | 1 |
| Clostridium_beijerinckii_BGS1_439 | 98.03 | I | 105 | 5880896 | 195648 | 10 |
| Clostridium_beijerinckii_DSM_53_516 | 97.64 | I | 346 | 5773247 | 40593 | 42 |
| Clostridium_beijerinckii_DSM_791_529 | 97.64 | I | 264 | 5781472 | 43059 | 41 |
| Clostridium_beijerinckii_G117_75 | 98.03 | I | 89 | 5811816 | 172488 | 14 |
| Clostridium_beijerinckii_HUN142_159 | 97.64 | I | 53 | 6106710 | 236200 | 8 |
| Clostridium_beijerinckii_NCIMB_14988_251 | 98.03 | I | 1 | 6485394 | 6485394 | 1 |
| Clostridium_beijerinckii_NCIMB_8052_8 | 97.64 | I | 1 | 6000632 | 6000632 | 1 |
| Clostridium_beijerinckii_NCP_260_520 | 98.03 | I | 242 | 5968330 | 59545 | 30 |
| Clostridium_beijerinckii_NRRL_B_528_526 | 97.64 | I | 233 | 6255488 | 70635 | 27 |
| Clostridium_beijerinckii_NRRL_B_591_521 | 97.24 | I | 358 | 5874824 | 39987 | 43 |
| Clostridium_beijerinckii_NRRL_B_593_522 | 96.85 | I | 305 | 6156662 | 46401 | 38 |
| Clostridium_beijerinckii_NRRL_B_596_528 | 96.46 | I | 393 | 6220133 | 38644 | 47 |
| Clostridium_beijerinckii_NRRL_B_598_138 | 97.24 | I | 1 | 6186879 | 6186879 | 1 |
| Clostridium_botulinum_111_249 | 98.82 | I | 1 | 3901300 | 3901300 | 1 |
| Clostridium_botulinum_12LNR10_CD_302 | 98.82 | I | 132 | 3041748 | 39896 | 22 |
| Clostridium_botulinum_12LNR13_CD_304 | 98.03 | I | 140 | 3075461 | 41782 | 24 |
| Clostridium_botulinum_12LNRI_CD_299 | 98.43 | I | 131 | 3005803 | 41798 | 21 |
| Clostridium_botulinum_202F_232 | 97.64 | I | 2 | 3914604 | 3874462 | 1 |
| Clostridium_botulinum_211_366 | 98.43 | I | 42 | 3712985 | 404531 | 4 |
| Clostridium_botulinum_246 | 98.43 | I | 1 | 3611897 | 3611897 | 1 |
| Clostridium_botulinum_277_00_250 | 99.21 | I | 90 | 3938115 | 133135 | 10 |
| Clostridium_botulinum_29401_CD_303 | 98.43 | I | 112 | 3046146 | 57518 | 18 |
| Clostridium_botulinum_38028_CD_301 | 98.43 | I | 104 | 3118863 | 48579 | 19 |
| Clostridium_botulinum_43243_CD_298 | 98.43 | I | 111 | 3005167 | 57521 | 16 |
| Clostridium_botulinum_47295_DC_309 | 99.21 | I | 108 | 3178512 | 51664 | 20 |
| Clostridium_botulinum_48212_CD_300 | 98.03 | I | 75 | 3005337 | 68539 | 14 |
| Clostridium_botulinum_49511_CD_307 | 98.03 | I | 85 | 3088897 | 64109 | 12 |
| Clostridium_botulinum_50867_CD_306 | 98.43 | I | 134 | 3077301 | 36158 | 22 |
| Clostridium_botulinum_51714_DC_308 | 98.82 | I | 101 | 3174715 | 59200 | 19 |
| Clostridium_botulinum_55741_CD_305 | 98.82 | I | 103 | 3040725 | 57521 | 17 |
| Clostridium_botulinum_58272_CD_313 | 98.82 | I | 128 | 3074857 | 40020 | 22 |
| Clostridium_botulinum_58752_CD_314 | 98.82 | I | 114 | 2825871 | 41221 | 20 |
| Clostridium_botulinum_69285_CD_310 | 98.82 | I | 146 | 2937195 | 61722 | 15 |
| Clostridium_botulinum_713_CBOT_288 | 98.43 | I | 191 | 3551054 | 41974 | 25 |
| Clostridium_botulinum_71840_CD_311 | 98.03 | I | 145 | 3001250 | 40406 | 22 |
| Clostridium_botulinum_A_str._ATCC_19397_9 | 98.82 | I | 1 | 3863450 | 3863450 | 1 |
| Clostridium_botulinum_A_str._ATCC_3502_19 | 99.21 | I | 2 | 3903260 | 3886916 | 1 |
| Clostridium_botulinum_A_str._Hall_10 | 98.82 | I | 1 | 3760560 | 3760560 | 1 |
| Clostridium_botulinum_A2_117_173 | 98.82 | I | 11 | 3808262 | 2752809 | 1 |
| Clostridium_botulinum_A2_str._Kyoto_18 | 98.82 | I | 1 | 4155278 | 4155278 | 1 |
| Clostridium_botulinum_A2B3_87_171 | 98.82 | I | 13 | 4168550 | 2767020 | 1 |
| Clostridium_botulinum_A2B7_92_172 | 98.82 | I | 14 | 4057812 | 698075 | 2 |
| Clostridium_botulinum_A3_str._Loch_Maree_13 | 99.21 | I | 2 | 4259691 | 3992906 | 1 |
| Clostridium_botulinum_AM1195_389 | 98.43 | I | 31 | 4271150 | 299090 | 6 |
| Clostridium_botulinum_AM1295_390 | 98.82 | I | 34 | 3900045 | 410269 | 3 |
| Clostridium_botulinum_AM370_391 | 98.82 | I | 31 | 4269660 | 398258 | 4 |
| Clostridium_botulinum_AM553_392 | 98.43 | I | 30 | 4308056 | 685346 | 3 |
| Clostridium_botulinum_An436_367 | 99.21 | I | 70 | 4201460 | 117448 | 11 |
| Clostridium_botulinum_ATCC_17786_322 | 98.43 | I | 61 | 3951940 | 193297 | 7 |
| Clostridium_botulinum_ATCC_17843_316 | 98.43 | I | 24 | 3906754 | 2083105 | 1 |
| Clostridium_botulinum_ATCC_17843_B5_365 | 98.43 | I | 79 | 3898546 | 116115 | 10 |
| Clostridium_botulinum_ATCC_17862_319 | 99.21 | I | 56 | 3907623 | 227875 | 6 |
| Clostridium_botulinum_ATCC_23387_317 | 98.03 | I | 29 | 3817674 | 790194 | 2 |
| Clostridium_botulinum_ATCC_25763_323 | 99.21 | I | 28 | 3889092 | 720423 | 3 |
| Clostridium_botulinum_ATCC_449_369 | 99.21 | I | 65 | 3777532 | 137365 | 10 |
| Clostridium_botulinum_ATCC_7949_320 | 98.82 | I | 35 | 3909427 | 612792 | 3 |
| Clostridium_botulinum_ATCC_9564_318 | 98.43 | I | 57 | 3813606 | 201673 | 7 |
| Clostridium_botulinum_B_str_Eklund_17BNRP14 | 8.43 | I | 2 | 3847969 | 3800327 | 1 |
| Clostridium_botulinum_B_str._Osaka05_137 | 98.03 | I | 3 | 4408349 | 4004744 | 1 |
| Clostridium_botulinum_B1_str._Okra_12 | 98.82 | I | 2 | 4107013 | 3958233 | 1 |
| Clostridium_botulinum_B2_128_174 | 98.82 | I | 10 | 3844467 | 2786970 | 1 |
| Clostridium_botulinum_B2_267_175 | 98.82 | I | 12 | 3903580 | 2837736 | 1 |
| Clostridium_botulinum_B2_275_170 | 98.82 | I | 13 | 3978188 | 2203691 | 1 |
| Clostridium_botulinum_B2_331_169 | 98.82 | I | 10 | 3809103 | 2732590 | 1 |
| Clostridium_botulinum_B2_433_177 | 98.82 | I | 12 | 4124526 | 2803320 | 1 |
| Clostridium_botulinum_B2_450_252 | 98.03 | I | 10 | 4320669 | 2916972 | 1 |
| Clostridium_botulinum_Ba4_657_16 | 98.82 | I | 3 | 4257769 | 3977794 | 1 |
| Clostridium_botulinum_Bf_39 | 97.64 | I | 70 | 4217754 | 170315 | 9 |
| Clostridium_botulinum_BKT015925_60 | 98.82 | I | 6 | 3207592 | 2773157 | 1 |
| Clostridium_botulinum_BKT028387_59 | 96.46 | I | 237 | 2833823 | 29150 | 33 |
| Clostridium_botulinum_C_D_str._BKT12695_168 | 98.43 | I | 89 | 2754780 | 63795 | 14 |
| Clostridium_botulinum_C_D_str._BKT2873_184 | 98.82 | I | 159 | 3165510 | 77101 | 14 |
| Clostridium_botulinum_C_D_str._BKT75002_185 | 98.82 | I | 121 | 3138765 | 77530 | 14 |
| Clostridium_botulinum_C_D_str._It1_188 | 98.43 | I | 77 | 2499706 | 59587 | 15 |
| Clostridium_botulinum_C_D_str._Sp77_187 | 98.43 | I | 148 | 3057314 | 40000 | 22 |
| Clostridium_botulinum_C_str._Eklund_40 | 95.67 | I | 76 | 2961186 | 145667 | 6 |
| Clostridium_botulinum_CB11_1_1_90 | 96.46 | I | 171 | 3823307 | 43401 | 26 |
| Clostridium_botulinum_CDC_1436_242 | 99.21 | I | 2 | 4365669 | 4089683 | 1 |
| Clostridium_botulinum_CDC_1632_470 | 98.82 | I | 1 | 4393047 | 4393047 | 1 |
| Clostridium_botulinum_CDC_1744_364 | 98.82 | I | 71 | 3959495 | 142985 | 8 |
| Clostridium_botulinum_CDC_5247_384 | 98.43 | I | 51 | 3839610 | 203210 | 5f |
| Clostridium_botulinum_CDC_53174_471 | 98.82 | I | 1 | 3867627 | 3867627 | 1 |
| Clostridium_botulinum_CDC_67190_468 | 98.82 | I | 3 | 4020063 | 3954777 | 1 |
| Clostridium_botulinum_CDC_69094_467 | 98.82 | I | 1 | 4089027 | 4089027 | 1 |
| Clostridium_botulinum_CDC_69096_503 | 98.43 | I | 26 | 4252578 | 3982791 | 1 |
| Clostridium_botulinum_CDC_795_381 | 98.82 | I | 97 | 3920281 | 96784 | 13 |
| Clostridium_botulinum_CDC28023_195 | 94.49 | I | 341 | 3966737 | 22262 | 51 |
| Clostridium_botulinum_CDC48719_194 | 91.73 | I | 310 | 3977994 | 26132 | 44 |
| Clostridium_botulinum_CDC66177_81 | 97.64 | I | 119 | 3852437 | 86296 | 15 |
| Clostridium_botulinum_CFSAN024410_230 | 98.82 | I | 131 | 4005128 | 59804 | 22 |
| Clostridium_botulinum_D_CCUG_7971_229 | 98.43 | I | 111 | 2808469 | 52759 | 17 |
| Clostridium_botulinum_D_str._16868_186 | 98.43 | I | 129 | 3083628 | 95198 | 12 |
| Clostridium_botulinum_DB_2_211 | 98.03 | I | 150 | 3915341 | 288306 | 5 |
| Clostridium_botulinum_E1_strBoNT_EBeluga_48 | 97.24 | I | 6 | 3999201 | 3863095 | 1 |
| Clostridium_botulinum_E3_str._Alaska_E43_15 | 98.43 | I | 1 | 3659644 | 3659644 | 1 |
| Clostridium_botulinum_F_357_176 | 98.43 | I | 15 | 3832122 | 957457 | 2 |
| Clostridium_botulinum_F_str._230613_20 | 93.31 | I | 2 | 4010614 | 3993083 | 1 |
| Clostridium_botulinum_F_str._Langeland_11 | 98.82 | I | 2 | 4012918 | 3995387 | 1 |
| Clostridium_botulinum_H04402_065_71 | 97.64 | I | 1 | 3919740 | 3919740 | 1 |
| Clostridium_botulinum_Hall_183_370 | 98.82 | I | 99 | 4002127 | 78163 | 16 |
| Clostridium_botulinum_Hall_4834_371 | 99.21 | I | 86 | 4025192 | 97924 | 11 |
| Clostridium_botulinum_Hall_80_378 | 98.82 | I | 65 | 3812410 | 175053 | 9 |
| Clostridium_botulinum_Hall_8388A_374 | 99.21 | I | 55 | 3909258 | 191646 | 7 |
| Clostridium_botulinum_Hall_8857Ab_372 | 99.21 | I | 84 | 3971034 | 101664 | 11 |
| Clostridium_botulinum_K15_393 | 98.43 | I | 114 | 3997935 | 100195 | 13 |
| Clostridium_botulinum_K3_394 | 98.43 | I | 218 | 3850229 | 221625 | 4 |
| Clostridium_botulinum_KAPB_3_210 | 98.43 | I | 128 | 3871084 | 726694 | 3 |
| Clostridium_botulinum_KF_Meyer_126_373 | 98.82 | I | 55 | 3891395 | 199059 | 7 |
| Clostridium_botulinum_KF_Meyer_33_380 | 98.82 | I | 98 | 3896775 | 72415 | 15 |
| Clostridium_botulinum_L_572_383 | 97.64 | I | 82 | 3766552 | 99892 | 11 |
| Clostridium_botulinum_LNC5_DC_312 | 99.21 | I | 83 | 2894028 | 55876 | 15 |
| Clostridium_botulinum_Mauritius_395 | 98.82 | I | 84 | 3869437 | 100079 | 13 |
| Clostridium_botulinum_McClung_844_379 | 98.82 | I | 48 | 3856611 | 219996 | 6 |
| Clostridium_botulinum_NCTC_2916_38 | 98.82 | I | 49 | 4031357 | 433501 | 3 |
| Clostridium_botulinum_NCTC_8550_247 | 98.43 | I | 1 | 3611898 | 3611898 | 1 |
| Clostridium_botulinum_Prevot_594_241 | 98.03 | I | 2 | 4334551 | 4077214 | 1 |
| Clostridium_botulinum_Prevot_697B_377 | 98.82 | I | 34 | 3791338 | 344362 | 4 |
| Clostridium_botulinum_Prevot_910_375 | 99.21 | I | 74 | 3942683 | 116912 | 10 |
| Clostridium_botulinum_Prevot_Dewping_376 | 98.82 | I | 42 | 3880168 | 252959 | 5 |
| Clostridium_botulinum_Prevot_Ped_1_385 | 98.03 | I | 81 | 3791027 | 125071 | 10 |
| Clostridium_botulinum_Prevot_R81_3A_386 | 98.43 | I | 64 | 3783752 | 200588 | 6 |
| Clostridium_botulinum_SU0634_420 | 98.82 | I | 43 | 3882852 | 201043 | 6 |
| Clostridium_botulinum_SU0635W_422 | 99.21 | I | 137 | 3945914 | 64297 | 16 |
| Clostridium_botulinum_SU0729_421 | 99.21 | I | 72 | 3929760 | 270392 | 6 |
| Clostridium_botulinum_SU0801_416 | 98.82 | I | 124 | 4210622 | 117792 | 10 |
| Clostridium_botulinum_SU0807 | 99.21 | I | 68 | 4091988 | 249852 | 6 |
| Clostridium_botulinum_SU0945_415 | 98.82 | I | 272 | 4534970 | 140479 | 11 |
| Clostridium_botulinum_SU0994_419 | 99.21 | I | 51 | 4296977 | 372385 | 5 |
| Clostridium_botulinum_SU0998_418 | 98.82 | I | 61 | 4136384 | 274519 | 5 |
| Clostridium_botulinum_SU1054_409 | 98.82 | I | 99 | 4311895 | 274519 | 5 |
| Clostridium_botulinum_SU1064_411 | 99.21 | I | 139 | 4368910 | 424584 | 4 |
| Clostridium_botulinum_SU1072_410 | 98.82 | I | 66 | 4281988 | 274519 | 4 |
| Clostridium_botulinum_SU1074_413 | 98.82 | I | 61 | 4055427 | 429106 | 4 |
| Clostridium_botulinum_SU1112_414 | 98.82 | I | 46 | 3929155 | 460210 | 3 |
| Clostridium_botulinum_SU1169_412 | 98.82 | I | 204 | 4495542 | 197058 | 8 |
| Clostridium_botulinum_SU1259_407 | 98.82 | I | 174 | 4175404 | 723257 | 2 |
| Clostridium_botulinum_SU1274_401 | 99.21 | I | 50 | 4230750 | 274519 | 6 |
| Clostridium_botulinum_SU1275_404 | 98.82 | I | 280 | 4305633 | 150850 | 7 |
| Clostridium_botulinum_SU1304_406 | 98.82 | I | 141 | 4306760 | 222901 | 6 |
| Clostridium_botulinum_SU1306_399 | 98.82 | I | 66 | 4267307 | 146794 | 11 |
| Clostridium_botulinum_SU1575NT_400 | 99.21 | I | 38 | 3873577 | 357116 | 4 |
| Clostridium_botulinum_SU1887_403 | 99.21 | I | 67 | 4010957 | 238528 | 4 |
| Clostridium_botulinum_SU1891_408 | 99.21 | I | 223 | 4476519 | 277699 | 5 |
| Clostridium_botulinum_SU1917_402 | 98.82 | I | 61 | 4274979 | 215880 | 5 |
| Clostridium_botulinum_SU1934_405 | 98.82 | I | 38 | 3990632 | 529942 | 2 |
| Clostridium_botulinum_SU1937_398 | 98.82 | I | 85 | 4285737 | 214161 | 5 |
| Clostridium_botulinum_type_C_BOTC | 97.64 | I | 135 | 2617336 | 145081 | 6 |
| Clostridium_botulinum_type_D_BOTD | 99.21 | I | 259 | 3059863 | 55039 | 18 |
| Clostridium_botulinum_V891_58 | 98.82 | I | 114 | 3172457 | 68878 | 16 |
| Clostridium_botulinum_VPI_7124_368 | 99.21 | I | 80 | 3864510 | 77273 | 16 |
| Clostridium_botulinum_Walls_8G_280 | 99.21 | I | 27 | 3978537 | 835326 | 2 |
| Clostridium_butyricum_5521_41 | 89.76 | I | 123 | 4540699 | 81014 | 17 |
| Clostridium_butyricum_60E.3_105 | 98.03 | I | 10 | 4644398 | 2372918 | 1 |
| Clostridium_butyricum_AGR2140_121 | 97.64 | I | 39 | 4550822 | 237318 | 7 |
| Clostridium_butyricum_CDC_51208_469 | 97.64 | I | 3 | 4639914 | 3809831 | 1 |
| Clostridium_butyricum_CWBI1009_255 | 97.64 | I | 340 | 4491619 | 26889 | 51 |
| Clostridium_butyricum_DKU_01_91 | 97.64 | I | 79 | 4519722 | 108221 | 13 |
| Clostridium_butyricum_DSM_10702_116 | 97.64 | I | 207 | 4596811 | 83186 | 17 |
| Clostridium_butyricum_E4_strBoNTEBL5262_52 | 97.64 | I | 13 | 4758422 | 757653 | 2 |
| Clostridium_butyricum_HM_68_253 | 97.64 | I | 2 | 4604758 | 3835983 | 1 |
| Clostridium_butyricum_JKY6D1_355 | 97.64 | I | 3 | 4618327 | 3819894 | 1 |
| Clostridium_butyricum_KNU_L09_352 | 97.64 | I | 2 | 4627894 | 3824894 | 1 |
| Clostridium_butyricum_TOA_434 | 97.64 | I | 3 | 4597202 | 3794139 | 1 |
| Clostridium_cadaveris_AGR2141_120 | 98.43 | I | 42 | 3542160 | 141803 | 8 |
| Clostridium_cadaveris_NLAE_zl_G419_543 | 98.82 | I | 52 | 3532192 | 120460 | 10 |
| Clostridium_carboxidivorans_P7_284 | 97.64 | I | 2 | 5752782 | 5732880 | 1 |
| Clostridium_cavendishii_DSM_21758_555 | 99.21 | I | 56 | 4987666 | 284556 | 7 |
| Clostridium_chauvoei_JF4335_1112 | 98.03 | I | 1 | 2887451 | 2887451 | 1 |
| Clostridium_chauvoeiCH3_CHAUPV | 98.03 | I | 309 | 2892977 | 72373 | 13 |
| Clostridium_chauvoeiCN3796_CHAU3796 | 98.03 | I | 292 | 2935638 | 79097 | 11 |
| Clostridium_colicanis_209318_97 | 99.21 | I | 10 | 3505620 | 2922022 | 1 |
| Clostridium_collagenovorans_DSM_3089_553 | 98.43 | I | 30 | 3482404 | 417404 | 3 |
| Clostridium_coskatii_PS02_430 | 98.03 | I | 85 | 4565108 | 130355 | 12 |
| Clostridium_coskatii_PTA_10522_437 | 97.64 | I | 112 | 4538837 | 90816 | 16 |
| Clostridium_disporicum_2789STDY5608827_335 | 98.03 | I | 110 | 3739749 | 84850 | 15 |
| Clostridium_disporicum_2789STDY5834855_330 | 97.64 | I | 119 | 3804830 | 80327 | 18 |
| Clostridium_disporicum_2789STDY5834856_333 | 98.03 | I | 57 | 3449564 | 144642 | 9 |
| Clostridium_drakei_SL1_160 | 97.64 | I | 122 | 5578774 | 151002 | 12 |
| Clostridium_estertheticum_sub_estertheticum_DSM8809 466 | 97.64 | I | 2 | 4785613 | 4760574 | 1 |
| Clostridium_fallax_DSM_2631_549 | 97.24 | I | 64 | 2747337 | 94344 | 9 |
| Clostridium_felsineum_DSM_794_524 | 98.03 | I | 100 | 5178654 | 177705 | 10 |
| Clostridium_haemolyticum_3629_HAEMOLYT | 97.64 | I | 285 | 2650828 | 55585 | 16 |
| Clostridium_haemolyticum_NCTC_8350_225 | 97.64 | I | 234 | 2465609 | 42678 | 18 |
| Clostridium_haemolyticum_NCTC_9693_180 | 97.64 | I | 125 | 2607656 | 55585 | 15 |
| Clostridium_hydrogeniformans_DSM_21757_163 | 99.21 | I | 31 | 4101506 | 314904 | 4 |
| Clostridium_intestinale_DSM_6191_554 | 98.82 | I | 33 | 4600598 | 368167 | 5 |
| Clostridium_intestinale_URNW_131 | 96.06 | I | 3 | 4677668 | 4667789 | 1 |
| Clostridium_kluyveri_DSM_555_7 | 98.03 | I | 2 | 4023800 | 3964618 | 1 |
| Clostridium_kluyveri_JZZ_472 | 97.24 | I | 2 | 4512934 | 4454353 | 1 |
| Clostridium_kluyveri_NBRC_12016_4 | 98.03 | I | 2 | 3955303 | 3896121 | 1 |
| Clostridium_ljungdahlii_DSM_13528_21 | 97.64 | I | 1 | 4630065 | 4630065 | 1 |
| Clostridium_ljungdahlii_DSM_13528_PETC_432 | 98.03 | I | 56 | 4572618 | 200112 | 8 |
| Clostridium_ljungdahlii_ERI_2_431 | 97.64 | I | 65 | 4363917 | 161324 | 7 |
| Clostridium_magnum_DSM_2767_429 | 98.43 | I | 25 | 6634930 | 740872 | 3 |
| Clostridium_novyi_A_str._4540_191 | 96.85 | I | 81 | 2500001 | 62812 | 12 |
| Clostridium_novyi_A_str._4552_228 | 98.03 | I | 119 | 2798065 | 47012 | 21 |
| Clostridium_novyi_A_str._4570_226 | 97.24 | I | 69 | 2324088 | 71398 | 9 |
| Clostridium_novyi_A_str._BKT29909_190 | 92.52 | I | 95 | 2463931 | 45649 | 17 |
| Clostridium_novyi_A_str._GD211209_189 | 96.06 | I | 104 | 2459310 | 38497 | 19 |
| Clostridium_novyi_A_str._NCTC_538_192 | 97.24 | I | 69 | 2519932 | 63662 | 14 |
| Clostridium_novyi_B_str._ATCC_27606_181 | 97.64 | I | 168 | 2613094 | 46394 | 20 |
| Clostridium_novyi_NT_6 | 99.21 | I | 1 | 2547720 | 2547720 | 1 |
| Clostridium_novyi_Type_BT46_oxer_NOVYIPV | 97.64 | I | 245 | 2606506 | 62864 | 15 |
| Clostridium_paraputrificum_2789STDY5834857_331 | 98.43 | I | 25 | 3597627 | 348122 | 4 |
| Clostridium_paraputrificum_373_A1_438 | 98.43 | I | 41 | 3488595 | 259466 | 5 |
| Clostridium_paraputrificum_AGR2156_119 | 98.43 | I | 30 | 3561289 | 320228 | 4 |
| Clostridium_pasteurianum_BC1_112 | 98.03 | I | 2 | 5044100 | 4990707 | 1 |
| Clostridium_pasteurianum_DSM_525_ATCC_6013_464 | 96.85 | I | 1 | 4352852 | 4352852 | 1 |
| Clostridium_pasteurianum_GL11_443 | 98.43 | I | 2 | 4677181 | 4485768 | 1 |
| Clostridium_pasteurianum_M150B_465 | 96.85 | I | 1 | 4351863 | 4351863 | 1 |
| Clostridium_perfringens_1207_CPER_287 | 99.21 | I | 95 | 3207161 | 85634 | 11 |
| Clostridium_perfringens_2789STDY5608889_338 | 98.82 | I | 48 | 3321076 | 2127284 | 1 |
| Clostridium_perfringens_ATCC_13124_5 | 98.82 | I | 1 | 3256683 | 3256683 | 1 |
| Clostridium_perfringens_B_str._ATCC_3626_43 | 93.7 | I | 98 | 3896305 | 88742 | 12 |
| Clostridium_perfringens_C_str._JGS1495_42 | 97.24 | I | 84 | 3661329 | 117588 | 10 |
| Clostridium_perfringens_CP4_343 | 98.82 | I | 98 | 3642209 | 83876 | 13 |
| Clostridium_perfringens_CPE_str._F4969_45 | 94.09 | I | 74 | 3510272 | 96499 | 10 |
| Clostridium_perfringens_E_str._JGS1987_44 | 92.91 | I | 101 | 4127102 | 88895 | 14 |
| Clostridium_perfringens_F262_72 | 98.82 | I | 14 | 3468406 | 3333039 | 1 |
| Clostridium_perfringens_FORC_003_aquarium_water_326 | 98.82 | I | 2 | 3395109 | 3338532 | 1 |
| Clostridium_perfringens_FORC_025_462 | 98.82 | I | 1 | 3343822 | 3343822 | 1 |
| Clostridium_perfringens_JFP718_473 | 98.82 | I | 56 | 3652220 | 197638 | 7 |
| Clostridium_perfringens_JFP727_476 | 99.21 | I | 47 | 3624033 | 151112 | 8 |
| Clostridium_perfringens_JFP728_475 | 99.21 | I | 85 | 3579626 | 83659 | 15 |
| Clostridium_perfringens_JFP771_481 | 98.43 | I | 81 | 3495308 | 118182 | 10 |
| Clostridium_perfringens_JFP774_474 | 98.43 | I | 56 | 3548595 | 142704 | 7 |
| Clostridium_perfringens_JFP795_477 | 98.43 | I | 67 | 3578912 | 114607 | 12 |
| Clostridium_perfringens_JFP796_480 | 99.21 | I | 114 | 3601148 | 75633 | 17 |
| Clostridium_perfringens_JFP801_478 | 99.21 | I | 59 | 3580610 | 143793 | 8 |
| Clostridium_perfringens_JFP804_479 | 98.43 | I | 117 | 3625968 | 71788 | 15 |
| Clostridium_perfringens_JFP810_482 | 98.82 | I | 95 | 3830406 | 161738 | 8 |
| Clostridium_perfringens_JFP826_483 | 98.82 | I | 70 | 3660408 | 105999 | 10 |
| Clostridium_perfringens_JFP828_484 | 98.82 | I | 54 | 3564370 | 125860 | 10 |
| Clostridium_perfringens_JFP829_485 | 98.82 | I | 108 | 3639686 | 71458 | 18 |
| Clostridium_perfringens_JFP833_486 | 98.43 | I | 96 | 3521770 | 84324 | 13 |
| Clostridium_perfringens_JFP834_487 | 98.82 | I | 121 | 3549140 | 96309 | 13 |
| Clostridium_perfringens_JFP836_488 | 98.82 | I | 51 | 3599532 | 163794 | 7 |
| Clostridium_perfringens_JFP914_489 | 99.21 | I | 79 | 3819232 | 104224 | 13 |
| Clostridium_perfringens_JFP916_490 | 98.82 | I | 67 | 3660641 | 160443 | 9 |
| Clostridium_perfringens_JFP921_491 | 99.21 | I | 78 | 3601767 | 100577 | 12 |
| Clostridium_perfringens_JFP922_492 | 98.82 | I | 76 | 3612099 | 186517 | 7 |
| Clostridium_perfringens_JFP923_493 | 98.82 | I | 48 | 3589715 | 212576 | 6 |
| Clostridium_perfringens_JFP941_494 | 98.82 | I | 64 | 3594640 | 109035 | 11 |
| Clostridium_perfringens_JFP961_495 | 99.21 | I | 66 | 3617219 | 138753 | 9 |
| Clostridium_perfringens_JFP978_496 | 99.21 | I | 68 | 3585277 | 106630 | 12 |
| Clostridium_perfringens_JFP980_497 | 98.82 | I | 67 | 3654588 | 108867 | 11 |
| Clostridium_perfringens_JFP981_498 | 99.21 | I | 68 | 3580367 | 131646 | 10 |
| Clostridium_perfringens_JFP982_499 | 98.82 | I | 62 | 3670557 | 137154 | 8 |
| Clostridium_perfringens_JFP983_500 | 98.82 | I | 46 | 3657522 | 204880 | 7 |
| Clostridium_perfringens_JFP986_502 | 99.21 | I | 58 | 3669049 | 158758 | 9 |
| Clostridium_perfringens_JFP99_501 | 98.82 | I | 70 | 3657868 | 174400 | 8 |
| Clostridium_perfringens_JJC_140 | 99.21 | I | 69 | 3259329 | 98246 | 9 |
| Clostridium_perfringens_JP55_423 | 98.43 | I | 6 | 3571229 | 3347300 | 1 |
| Clostridium_perfringens_JP838_424 | 98.82 | I | 5 | 4070811 | 3530414 | 1 |
| Clostridium_perfringens_MJR7757A_361 | 98.43 | I | 228 | 3590266 | 51972 | 22 |
| Clostridium_perfringens_NCTC8239_str._46 | 95.28 | I | 55 | 3324319 | 134604 | 6 |
| Clostridium_perfringens_str._13_3 | 98.03 | I | 2 | 3085740 | 3031430 | 1 |
| Clostridium_perfringens_Type_A_PERACSIR | 98.82 | I | 228 | 3483931 | 226748 | 4 |
| Clostridium_perfringens_Type_A1491_PERA1491 | 98.82 | I | 140 | 3279841 | 2055711 | 1 |
| Clostridium_perfringens_Type_B1240_PERB1240 | 98.82 | I | 122 | 3547357 | 261573 | 3 |
| Clostridium_perfringens_Type_B3424_PERB3424 | 98.82 | I | 243 | 3732983 | 410377 | 3 |
| Clostridium_perfringens_Type_C883_PERC883 | 99.21 | I | 282 | 3683980 | 291793 | 4 |
| Clostridium_perfringens_Type_D3688_PERD3683 | 98.82 | I | 166 | 3687986 | 238842 | 4 |
| Clostridium_perfringens_Type_DSB170D_PERDPV | 98.82 | I | 115 | 3495957 | 237771 | 4 |
| Clostridium_perfringens_WAL_14572_68 | 98.43 | I | 20 | 3466039 | 2084089 | 1 |
| Clostridium_proteolyticum_DSM_3090_557 | 97.24 | I | 45 | 2818024 | 121571 | 8 |
| Clostridium_puniceum_DSM_2619_523 | 98.03 | I | 245 | 6082167 | 68127 | 31 |
| Clostridium_ragsdalei_P11_436 | 97.64 | I | 79 | 4424992 | 119012 | 11 |
| Clostridium_roseum_DSM_6424_515 | 97.64 | I | 262 | 4944863 | 49535 | 32 |
| Clostridium_roseum_DSM_7320_517 | 98.03 | I | 124 | 5067725 | 86225 | 18 |
| Clostridium_saccharobutylicum_BAS_B3_SW_136_508 | 97.64 | I | 1 | 5108304 | 5108304 | 1 |
| Clostridium_saccharobutylicum_DSM_13864_133 | 98.03 | I | 1 | 5107814 | 5107814 | 1 |
| Clostridium_saccharobutylicum_L1_8_514 | 98.43 | I | 16 | 5173344 | 482513 | 4 |
| Clostridium_saccharobutylicum_NCP_165_519 | 98.43 | I | 142 | 4900327 | 83530 | 16 |
| Clostridium_saccharobutylicum_NCP_195_511 | 97.64 | I | 1 | 5108176 | 5108176 | 1 |
| Clostridium_saccharobutylicum_NCP_200_506 | 98.03 | I | 1 | 5108287 | 5108287 | 1 |
| Clostridium_saccharobutylicum_NCP_258_510 | 97.64 | I | 1 | 4950933 | 4950933 | 1 |
| Clostridium_saccharoperbutylacetonicum_N1_4HMT_87 | 97.24 | I | 2 | 6666445 | 6530257 | 1 |
| Clostridium_saccharoperbutylacetonicum_N1_ATCC27021_86 | 95.67 | I | 210 | 6569628 | 78354 | 30 |
| Clostridium_saccharoperbutylacetonicum_N1_504_507 | 97.24 | I | 2 | 6219394 | 6216458 | 1 |
| Clostridium_sartagoforme_AAU1_114 | 94.09 | I | 323 | 3983371 | 25206 | 45 |
| Clostridium_saudiense_JCC_147 | 97.64 | I | 100 | 3653762 | 89852 | 13 |
| Clostridium_scatologenes_ATCC_25775_278 | 97.24 | I | 1 | 5749410 | 5749410 | 1 |
| Clostridium_senegalense_JC122_type_strain_JC122_76 | 95.67 | I | 83 | 3925888 | 721927 | 2 |
| Clostridium_septicum_P1044_537 | 97.64 | I | 79 | 3298970 | 71267 | 16 |
| Clostridium_septicum38_858V_SEPTPV | 98.43 | I | 342 | 3373031 | 50613 | 21 |
| Clostridium_septicumCN3204_SEP3204B | 98.43 | I | 218 | 3344920 | 52620 | 20 |
| Clostridium_septicumCN368_SEPT368 | 98.43 | I | 243 | 3308673 | 44576 | 21 |
| Clostridium_sp._7_2_43FAA_34 | 98.03 | I | 5 | 3813122 | 3226588 | 1 |
| Clostridium_sp._Ade.TY_135 | 98.03 | I | 66 | 3113901 | 139705 | 7 |
| Clostridium_sp._ATCC_25772_397 | 98.43 | I | 241 | 4009413 | 62088 | 18 |
| Clostridium_sp._BL_8_531 | 97.64 | I | 231 | 6045940 | 56741 | 35 |
| Clostridium_sp._C8_282 | 97.24 | I | 125 | 4020958 | 59667 | 16 |
| Clostridium_sp._CL_6_212 | 98.43 | I | 17 | 4325182 | 695847 | 2 |
| Clostridium_sp._CL_6_human_stool_245 | 98.43 | I | 17 | 4325182 | 695847 | 2 |
| Clostridium_sp._DL_VIII_62 | 98.43 | I | 1 | 6477357 | 6477357 | 1 |
| Clostridium_sp._HMSC19A10_461 | 97.64 | I | 100 | 4538577 | 125680 | 11 |
| Clostridium_sp._IBUN125C_276 | 97.64 | I | 71 | 4596888 | 133994 | 10 |
| Clostridium_sp._IBUN13A_277 | 96.46 | I | 261 | 4643590 | 42668 | 29 |
| Clostridium_sp._IBUN22A_275 | 95.67 | I | 208 | 4607385 | 41410 | 33 |
| Clostridium_sp._IBUN62F_274 | 97.64 | I | 85 | 3836807 | 120226 | 11 |
| Clostridium_sp._L74_321 | 98.82 | I | 81 | 3687211 | 123171 | 9 |
| Clostridium_sp._LF2_214 | 98.82 | I | 15 | 3750216 | 2057217 | 1 |
| Clostridium_sp._Marseille_P2414_534 | 99.21 | I | 4 | 3799489 | 3796548 | 1 |
| Clostridium_sp._Marseille_P2434_536 | 98.43 | I | 5 | 3083338 | 2631890 | 1 |
| Clostridium_sp._ND2_329 | 98.82 | I | 11 | 3732966 | 2114283 | 1 |
| Clostridium_sporogenes_1961_2_345 | 98.43 | I | 40 | 4092065 | 213496 | 5 |
| Clostridium_sporogenes_1990_344 | 98.43 | I | 59 | 4163429 | 187341 | 7 |
| Clostridium_sporogenes_2007_351 | 98.43 | I | 33 | 4170948 | 321923 | 4 |
| Clostridium_sporogenes_66_CBOT_289 | 98.03 | I | 250 | 4220556 | 35618 | 36 |
| Clostridium_sporogenes_8_O_428 | 98.03 | I | 19 | 4088089 | 929532 | 2 |
| Clostridium_sporogenes_87_0535_260 | 98.03 | I | 202 | 4021062 | 46656 | 27 |
| Clostridium_sporogenes_88_0163_261 | 96.85 | I | 213 | 4101907 | 40700 | 31 |
| Clostridium_sporogenes_ATCC_15579_30 | 98.43 | I | 2 | 4102325 | 2710723 | 1 |
| Clostridium_sporogenes_ATCC_19404_396 | 98.03 | I | 48 | 4066128 | 344087 | 5 |
| Clostridium_sporogenes_DSM_795_283 | 98.03 | I | 1 | 4142990 | 4142990 | 1 |
| Clostridium_sporogenes_NCIMB_10696_279 | 98.03 | I | 1 | 4141984 | 4141984 | 1 |
| Clostridium_sporogenes_PA_3679_1961_4_346 | 98.43 | I | 85 | 3968020 | 115686 | 11 |
| Clostridium_sporogenes_PA_3679_67 | 98.43 | I | 107 | 4180273 | 172248 | 8 |
| Clostridium_sporogenes_PA_3679_Camp_350 | 98.82 | I | 57 | 3984395 | 159201 | 9 |
| Clostridium_sporogenes_PA_3679_FDA_348 | 98.82 | I | 86 | 4021963 | 151708 | 10 |
| Clostridium_sporogenes_PA_3679_NFL_349 | 98.43 | I | 79 | 3959602 | 136601 | 12 |
| Clostridium_sporogenes_PA_3679_UW_347 | 98.82 | I | 48 | 3926962 | 150182 | 10 |
| Clostridium_sporogenes_UC9000_324 | 97.64 | I | 111 | 4338649 | 110240 | 13 |
| Clostridium_sulfidigenes_113A_207 | 95.67 | I | 96 | 3717420 | 48174 | 26 |
| Clostridium_tepidiprofundi_DSM_19306_427 | 98.03 | I | 175 | 3060113 | 65328 | 13 |
| Clostridium_tetani_184.08_235 | 96.85 | I | 152 | 2914338 | 70366 | 14 |
| Clostridium_tetani_3911_TET3911 | 98.82 | I | 107 | 2874950 | 263604 | 4 |
| Clostridium_tetani_A_243 | 98.43 | I | 93 | 2824295 | 96331 | 10 |
| Clostridium_tetani_ATCC_19406_217 | 98.82 | I | 50 | 2789793 | 181872 | 5 |
| Clostridium_tetani_ATCC_453_219 | 98.43 | I | 40 | 2890535 | 253841 | 6 |
| Clostridium_tetani_ATCC_454_281 | 98.03 | I | 67 | 2852621 | 87223 | 11 |
| Clostridium_tetani_ATCC_9441_218 | 98.43 | I | 28 | 2800144 | 1734923 | 1 |
| Clostridium_tetani_C2_220 | 98.82 | I | 35 | 2829019 | 263508 | 4 |
| Clostridium_tetani_CN655_234 | 98.43 | I | 118 | 2850319 | 110001 | 9 |
| Clostridium_tetani_E88_Massachusetts | 98.43 | I | 2 | 2873333 | 2799251 | 1 |
| Clostridium_tunisiense_TJ_78 | 85.83 | I | 60 | 4308385 | 341305 | 5 |
| Clostridium_tyrobutyricum_DIVETGP_148 | 98.43 | I | 45 | 3018999 | 168670 | 6 |
| Clostridium_tyrobutyricum_DSM2637_ATCC25755_124 | 98.03 | I | 44 | 3022704 | 133576 | 8 |
| Clostridium_tyrobutyricum_FAM22552_238 | 98.43 | I | 58 | 3052855 | 166858 | 7 |
| Clostridium_tyrobutyricum_FAM22553_239 | 98.03 | I | 62 | 3085051 | 187297 | 6 |
| Clostridium_tyrobutyricum_IFP923_359 | 98.03 | I | 139 | 3190249 | 69218 | 14 |
| Clostridium_tyrobutyricum_KCTC_5387_433 | 98.03 | I | 2 | 3134437 | 3071606 | 1 |
| Clostridium_tyrobutyricum_UC7086_85 | 98.03 | I | 110 | 3064215 | 70370 | 14 |
| Clostridium_ventriculi_17_358 (Sarcina) | 96.46 | I | 32 | 2479673 | 260452 | 3 |
| Clostridium_ventriculi_2789STDY5834858_332 (Sarcina) | 96.46 | I | 31 | 2457263 | 342082 | 3 |
| Clostridium_ventriculi_357 (Sarcina) | 96.06 | I | 91 | 2474230 | 74523 | 11 |
| Clostridium_cellobioparum_DSM_1351_ATCC_15832_158 | 99.21 | III | 80 | 6132222 | 194099 | 12 |
| Clostridium_cellulolyticum_H10_17 | 98.03 | III | 1 | 4068724 | 4068724 | 1 |
| Clostridium_clariflavum_4_2a_141 | 99.21 | III | 4 | 4872398 | 4415514 | 1 |
| Clostridium_clariflavum_DSM_19732_66 | 99.21 | III | 1 | 4897678 | 4897678 | 1 |
| Clostridium_josui_JCM_17888_143 | 98.82 | III | 2 | 4469680 | 3589078 | 1 |
| Clostridium_papyrosolvens_DSM_2782_50 | 97.64 | III | 31 | 4915287 | 279057 | 6 |
| Clostridium_sp._Bc_iso_3_444 | 98.82 | III | 4 | 4327139 | 4269335 | 1 |
| Clostridium_sp._BNL1100_69 | 98.43 | III | 1 | 4613747 | 4613747 | 1 |
| Clostridium_stercorarium_sub._leptospartum_DSM9219_442 | 98.03 | III | 1 | 3148357 | 3148357 | 1 |
| Clostridium_stercorarium_sub._stercorarium_DSM_8532_88 | 98.03 | III | 1 | 2970010 | 2970010 | 1 |
| Clostridium_stercorarium_sub. thermolacticum_DSM2910_441 | 98.03 | III | 1 | 3035622 | 2970010 | 1 |
| Clostridium_termitidis_CT1112_89 | 98.43 | III | 78 | 6415858 | 146289 | 15 |
| Clostridium_leptum_DSM_753_25 | 97.64 | iv /XIVa | 21 | 3270209 | 452649 | 4 |
| Clostridium_sporosphaeroides_DSM_1294_VPI_4527_111 | 97.24 | IV/XIVa | 21 | 3174421 | 324437 | 4 |
| Clostridioides_difficile_VL_0092_988 | 98.82 | XIa | 321 | 4157070 | 90393 | 16 |
| Clostridium_difficile_002_P50_2011_574 | 98.82 | XIa | 79 | 4103061 | 207265 | 8 |
| Clostridium_difficile_01A09CD0020_822 | 98.43 | XIa | 103 | 4211492 | 116608 | 13 |
| Clostridium_difficile_050_P50_2011_576 | 98.43 | XIa | 65 | 4227225 | 211060 | 8 |
| Clostridium_difficile_08ACD0030_812 | 98.43 | XIa | 1 | 4167076 | 4167076 | 1 |
| Clostridium_difficile_103_815 | 98.03 | XIa | 162 | 4012303 | 131308 | 9 |
| Clostridium_difficile_106_814 | 98.03 | XIa | 106 | 4044391 | 137376 | 10 |
| Clostridium_difficile_133_816 | 98.03 | XIa | 150 | 4152605 | 137573 | 10 |
| Clostridium_difficile_20100211_824 | 99.21 | XIa | 5 | 4189946 | 2845247 | 1 |
| Clostridium_difficile_20100502_825 | 98.82 | XIa | 4 | 4201508 | 3853484 | 1 |
| Clostridium_difficile_20110270_833 | 99.21 | XIa | 4 | 4179937 | 3881918 | 1 |
| Clostridium_difficile_20110740_834 | 98.82 | XIa | 9 | 4279635 | 3058973 | 1 |
| Clostridium_difficile_20110995_836 | 98.82 | XIa | 2 | 4092490 | 3015771 | 1 |
| Clostridium_difficile_20111003_837 | 98.82 | XIa | 3 | 4200115 | 2285964 | 1 |
| Clostridium_difficile_20111144_831 | 98.43 | XIa | 13 | 4259046 | 2732843 | 1 |
| Clostridium_difficile_20121412_842 | 98.82 | XIa | 4 | 4278990 | 2484966 | 1 |
| Clostridium_difficile_22_1_820 | 98.43 | XIa | 58 | 4180898 | 196766 | 7 |
| Clostridium_difficile_5_3_790 | 98.82 | XIa | 27 | 4009318 | 786725 | 2 |
| Clostridium_difficile_6042_636 | 81.89 | XIa | 314 | 3945922 | 24692 | 46 |
| Clostridium_difficile_630_561 | 98.82 | XIa | 2 | 4298133 | 4290252 | 1 |
| Clostridium_difficile_630Derm_800 | 98.82 | XIa | 1 | 4293049 | 4293049 | 1 |
| Clostridium_difficile_655_631 | 96.85 | XIa | 207 | 4081576 | 36927 | 35 |
| Clostridium_difficile_7_10492_818 | 98.43 | XIa | 74 | 4317574 | 215323 | 7 |
| Clostridium_difficile_848 | 98.82 | XIa | 159 | 4051221 | 96828 | 14 |
| Clostridium_difficile_BR81_843 | 98.43 | XIa | 1 | 4124384 | 4124384 | 1 |
| Clostridium_difficile_CD03_1454 | 99.21 | XIa | 53 | 3841898 | 147309 | 9 |
| Clostridium_difficile_CD04_1396 | 98.03 | XIa | 61 | 4254923 | 170309 | 9 |
| Clostridium_difficile_CD05_1411 | 98.82 | XIa | 34 | 4443217 | 277295 | 4 |
| Clostridium_difficile_CD06_1403 | 98.82 | XIa | 42 | 4127790 | 231706 | 6 |
| Clostridium_difficile_CD07_1422 | 98.43 | XIa | 32 | 4132581 | 246756 | 7 |
| Clostridium_difficile_CD08_1449 | 98.43 | XIa | 53 | 4224446 | 199231 | 7 |
| Clostridium_difficile_CD10_165_808 | 98.82 | XIa | 67 | 4277571 | 210313 | 7 |
| Clostridium_difficile_CD105HE1_849 | 98.03 | XIa | 46 | 4153465 | 230007 | 7 |
| Clostridium_difficile_CD105KSE2_1198 | 98.43 | XIa | 122 | 4228132 | 180336 | 9 |
| Clostridium_difficile_CD105KSE3_1199 | 98.82 | XIa | 189 | 4295063 | 161100 | 9 |
| Clostridium_difficile_CD105KSE5_1197 | 98.82 | XIa | 378 | 4395161 | 277022 | 6 |
| Clostridium_difficile_CD105KSE6_1196 | 98.82 | XIa | 124 | 4262632 | 190962 | 7 |
| Clostridium_difficile_CD105KSO8_1201 | 98.82 | XIa | 90 | 4521395 | 221277 | 6 |
| Clostridium_difficile_CD111_700 | 95.28 | XIa | 240 | 4047257 | 35117 | 38 |
| Clostridium_difficile_CD113_701 | 97.64 | XIa | 127 | 4255649 | 82434 | 18 |
| Clostridium_difficile_CD12_1446 | 98.82 | XIa | 23 | 4076947 | 821395 | 2 |
| Clostridium_difficile_CD13_1445 | 98.82 | XIa | 39 | 4132205 | 229911 | 6 |
| Clostridium_difficile_CD131_608 | 96.46 | XIa | 134 | 4320519 | 57300 | 27 |
| Clostridium_difficile_CD144_611 | 98.43 | XIa | 56 | 4095302 | 184803 | 8 |
| Clostridium_difficile_CD15_1452 | 98.82 | XIa | 52 | 4155465 | 209526 | 6 |
| Clostridium_difficile_CD159_613 | 96.06 | XIa | 290 | 3986487 | 24265 | 52 |
| Clostridium_difficile_CD166_616 | 98.82 | XIa | 56 | 4289366 | 222760 | 8 |
| Clostridium_difficile_CD18_1435 | 98.43 | XIa | 39 | 4142242 | 246977 | 5 |
| Clostridium_difficile_CD19_1458 | 98.82 | XIa | 62 | 4022263 | 140793 | 10 |
| Clostridium_difficile_CD196_562 | 98.82 | XIa | 1 | 4110554 | 4110554 | 1 |
| Clostridium_difficile_CD21_1414 | 98.43 | XIa | 43 | 4125071 | 229812 | 6 |
| Clostridium_difficile_CD211_628 | 98.03 | XIa | 187 | 4054786 | 42827 | 30 |
| Clostridium_difficile_CD212_629 | 98.43 | XIa | 36 | 4003929 | 240914 | 6 |
| Clostridium_difficile_CD22_1423 | 98.82 | XIa | 35 | 4178020 | 247845 | 5 |
| Clostridium_difficile_CD23_1419 | 98.43 | XIa | 38 | 4061847 | 263841 | 5 |
| Clostridium_difficile_CD24_1407 | 98.43 | XIa | 44 | 4168771 | 204915 | 7 |
| Clostridium_difficile_CD25_1412 | 98.82 | XIa | 41 | 4293106 | 228970 | 7 |
| Clostridium_difficile_CD26_1388 | 98.82 | XIa | 26 | 4014727 | 624690 | 3 |
| Clostridium_difficile_CD26A54_R_809 | 98.82 | XIa | 4 | 4146282 | 1881735 | 2 |
| Clostridium_difficile_CD26A54_S_810 | 99.21 | XIa | 6 | 4166728 | 1445886 | 2 |
| Clostridium_difficile_CD27_1391 | 98.43 | XIa | 41 | 4197119 | 265736 | 7 |
| Clostridium_difficile_CD28_1392 | 98.43 | XIa | 54 | 4157621 | 201745 | 7 |
| Clostridium_difficile_CD30_1400 | 98.03 | XIa | 47 | 4124166 | 188065 | 9 |
| Clostridium_difficile_CD31_1437 | 98.43 | XIa | 34 | 4236825 | 283093 | 5 |
| Clostridium_difficile_CD35_1438 | 98.82 | XIa | 39 | 4025541 | 264934 | 4 |
| Clostridium_difficile_CD39_1432 | 98.82 | XIa | 30 | 4251978 | 402273 | 3 |
| Clostridium_difficile_CD41_1448 | 98.82 | XIa | 30 | 4181594 | 438667 | 4 |
| Clostridium_difficile_CD42_1416 | 98.43 | XIa | 28 | 4307274 | 476381 | 3 |
| Clostridium_difficile_CD43_1428 | 98.43 | XIa | 54 | 3909083 | 141853 | 10 |
| Clostridium_difficile_CD44_1431 | 98.43 | XIa | 49 | 4429426 | 216355 | 6 |
| Clostridium_difficile_CD45_597 | 98.03 | XIa | 176 | 4367906 | 54395 | 26 |
| Clostridium_difficile_CD46_1447 | 98.82 | XIa | 24 | 4077061 | 821490 | 2 |
| Clostridium_difficile_CD49_1401 | 98.82 | XIa | 59 | 4208917 | 245737 | 6 |
| Clostridium_difficile_CD51_1417 | 98.43 | XIa | 41 | 4215495 | 274737 | 6 |
| Clostridium_difficile_CD52_1420 | 98.43 | XIa | 47 | 4417523 | 212914 | 6 |
| Clostridium_difficile_CD53_1390 | 98.43 | XIa | 25 | 4079056 | 823366 | 2 |
| Clostridium_difficile_CD54_1402 | 98.82 | XIa | 34 | 4214944 | 633620 | 3 |
| Clostridium_difficile_CD57_1444 | 98.82 | XIa | 34 | 4313723 | 300939 | 4 |
| Clostridium_difficile_CD58_1427 | 98.82 | XIa | 32 | 4196484 | 383688 | 4 |
| Clostridium_difficile_CD60_1399 | 98.43 | XIa | 54 | 4122619 | 192092 | 9 |
| Clostridium_difficile_CD62_1397 | 98.82 | XIa | 27 | 4067368 | 405787 | 3 |
| Clostridium_difficile_CD63_1387 | 98.43 | XIa | 32 | 4012749 | 262729 | 4 |
| Clostridium_difficile_CD64_1425 | 98.43 | XIa | 38 | 4154464 | 268013 | 6 |
| Clostridium_difficile_CD65_1393 | 98.82 | XIa | 39 | 4101999 | 247554 | 6 |
| Clostridium_difficile_CD66_1406 | 98.43 | XIa | 28 | 4068751 | 406103 | 3 |
| Clostridium_difficile_CD67_1405 | 99.21 | XIa | 55 | 3961761 | 153556 | 8 |
| Clostridium_difficile_CD68_602 | 90.55 | XIa | 141 | 3975975 | 53580 | 22 |
| Clostridium_difficile_CD69_1395 | 98.82 | XIa | 27 | 4079436 | 357688 | 3 |
| Clostridium_difficile_CD70_1451 | 98.43 | XIa | 33 | 4074098 | 230885 | 6 |
| Clostridium_difficile_CD8_15_813 | 98.82 | XIa | 44 | 4249791 | 235991 | 5 |
| Clostridium_difficile_CD8_586 | 96.46 | XIa | 280 | 4044267 | 28010 | 48 |
| Clostridium_difficile_CD9_587 | 98.82 | XIa | 87 | 4310458 | 685892 | 3 |
| Clostridium_difficile_CD90_697 | 97.64 | XIa | 123 | 4029007 | 74899 | 16 |
| Clostridium_difficile_DA00114_641 | 96.85 | XIa | 165 | 4058203 | 46799 | 27 |
| Clostridium_difficile_DA00126_642 | 94.49 | XIa | 91 | 4167768 | 115337 | 12 |
| Clostridium_difficile_DA00128_643 | 96.46 | XIa | 95 | 4217009 | 88258 | 15 |
| Clostridium_difficile_DA00132_646 | 97.64 | XIa | 78 | 4070905 | 99187 | 14 |
| Clostridium_difficile_DA00134_647 | 98.43 | XIa | 98 | 4115079 | 107219 | 11 |
| Clostridium_difficile_DA00141_648 | 98.82 | XIa | 52 | 4089728 | 130697 | 10 |
| Clostridium_difficile_DA00142_649 | 98.03 | XIa | 193 | 4183071 | 44831 | 33 |
| Clostridium_difficile_DA00145_650 | 97.64 | XIa | 132 | 4051066 | 55379 | 25 |
| Clostridium_difficile_DA00154_652 | 98.43 | XIa | 102 | 4126663 | 109889 | 14 |
| Clostridium_difficile_DA00160_653 | 98.82 | XIa | 98 | 4134851 | 113054 | 13 |
| Clostridium_difficile_DA00183_656 | 98.82 | XIa | 114 | 4071468 | 67760 | 20 |
| Clostridium_difficile_DA00193_658 | 82.68 | XIa | 60 | 3969329 | 141166 | 9 |
| Clostridium_difficile_DA00195_659 | 98.43 | XIa | 95 | 4108697 | 95853 | 16 |
| Clostridium_difficile_DA00196_660 | 98.82 | XIa | 22 | 4254880 | 623272 | 3 |
| Clostridium_difficile_DA00197_661 | 98.82 | XIa | 18 | 4122528 | 451615 | 4 |
| Clostridium_difficile_DA00211_664 | 98.82 | XIa | 90 | 4072757 | 88029 | 16 |
| Clostridium_difficile_DA00238_669 | 97.64 | XIa | 94 | 4163916 | 96856 | 14 |
| Clostridium_difficile_DA00244_670 | 98.82 | XIa | 15 | 4067645 | 761856 | 2 |
| Clostridium_difficile_DA00245_671 | 98.03 | XIa | 336 | 3985935 | 20188 | 61 |
| Clostridium_difficile_DA00273_607 | 98.43 | XIa | 271 | 4095984 | 25512 | 47 |
| Clostridium_difficile_DA00307_675 | 97.64 | XIa | 219 | 4147275 | 36095 | 37 |
| Clostridium_difficile_DA00313_677 | 98.82 | XIa | 112 | 4083557 | 70240 | 20 |
| Clostridium_difficile_E1_789 | 99.21 | XIa | 212 | 3945725 | 38456 | 31 |
| Clostridium_difficile_E12_782 | 98.82 | XIa | 353 | 4009421 | 22652 | 55 |
| Clostridium_difficile_E13_773 | 98.82 | XIa | 262 | 4146240 | 41362 | 31 |
| Clostridium_difficile_E14_767 | 98.82 | XIa | 319 | 4131267 | 32211 | 40 |
| Clostridium_difficile_E23_766 | 98.82 | XIa | 264 | 4015392 | 37968 | 34 |
| Clostridium_difficile_E9_786 | 98.82 | XIa | 373 | 4156306 | 29903 | 41 |
| Clostridium_difficile_F480_748 | 98.82 | XIa | 32 | 3989604 | 241744 | 6 |
| Clostridium_difficile_F548_750 | 98.82 | XIa | 17 | 4257706 | 695337 | 3 |
| Clostridium_difficile_F601_751 | 98.82 | XIa | 40 | 4103199 | 214607 | 8 |
| Clostridium_difficile_G46_794 | 99.21 | XIa | 1 | 4189317 | 4189317 | 1 |
| Clostridium_difficile_H3_819 | 98.82 | XIa | 76 | 4138192 | 217879 | 7 |
| Clostridium_difficile_KY62_802 | 98.43 | XIa | 277 | 4040078 | 187206 | 7 |
| Clostridium_difficile_KY64_803 | 98.43 | XIa | 324 | 4035637 | 180982 | 7 |
| Clostridium_difficile_LIBA_5784_797 | 98.43 | XIa | 44 | 4156825 | 234855 | 6 |
| Clostridium_difficile_M68_572 | 98.43 | XIa | 1 | 4308325 | 4308325 | 1 |
| Clostridium_difficile_NAP07_571 | 98.03 | XIa | 33 | 3905466 | 525841 | 4 |
| Clostridium_difficile_P1_687 | 98.82 | XIa | 160 | 4104507 | 48238 | 28 |
| Clostridium_difficile_P21_674 | 98.43 | XIa | 53 | 4090537 | 162302 | 9 |
| Clostridium_difficile_P32_726 | 98.43 | XIa | 44 | 4027255 | 237219 | 7 |
| Clostridium_difficile_P33_755 | 98.03 | XIa | 57 | 4103431 | 150350 | 9 |
| Clostridium_difficile_P36_728 | 98.82 | XIa | 40 | 4048210 | 233587 | 8 |
| Clostridium_difficile_P37_757 | 98.43 | XIa | 62 | 4080614 | 180173 | 8 |
| Clostridium_difficile_P41_756 | 98.82 | XIa | 62 | 4119865 | 234033 | 6 |
| Clostridium_difficile_P42_729 | 98.82 | XIa | 53 | 4185941 | 195731 | 7 |
| Clostridium_difficile_P46_731 | 98.82 | XIa | 60 | 4125436 | 196884 | 9 |
| Clostridium_difficile_P48_732 | 98.43 | XIa | 68 | 4172979 | 157205 | 9 |
| Clostridium_difficile_P49_733 | 98.82 | XIa | 46 | 4153887 | 235599 | 8 |
| Clostridium_difficile_P5_689 | 98.82 | XIa | 181 | 4048946 | 44667 | 32 |
| Clostridium_difficile_P50_734 | 98.82 | XIa | 29 | 4395649 | 486408 | 4 |
| Clostridium_difficile_P51_735 | 98.82 | XIa | 56 | 4256776 | 161476 | 10 |
| Clostridium_difficile_P53_758 | 98.82 | XIa | 41 | 4025043 | 236448 | 8 |
| Clostridium_difficile_P6_673 | 95.67 | XIa | 167 | 4167114 | 48839 | 28 |
| Clostridium_difficile_P64_759 | 97.24 | XIa | 72 | 4226724 | 184824 | 8 |
| Clostridium_difficile_P68_760 | 97.64 | XIa | 49 | 4056190 | 246498 | 7 |
| Clostridium_difficile_P69_739 | 98.82 | XIa | 49 | 4213133 | 228563 | 8 |
| Clostridium_difficile_P70_740 | 98.43 | XIa | 48 | 4049371 | 193767 | 8 |
| Clostridium_difficile_P71_742 | 98.82 | XIa | 52 | 4130145 | 163133 | 8 |
| Clostridium_difficile_P72_741 | 98.82 | XIa | 44 | 4057420 | 207373 | 7 |
| Clostridium_difficile_P75_745 | 98.82 | XIa | 54 | 4050593 | 191288 | 9 |
| Clostridium_difficile_P77_746 | 98.03 | XIa | 61 | 4155874 | 188876 | 8 |
| Clostridium_difficile_P78_738 | 98.43 | XIa | 60 | 4119601 | 181575 | 8 |
| Clostridium_difficile_P8_715 | 93.7 | XIa | 203 | 4292666 | 44870 | 28 |
| Clostridium_difficile_QCD_37x79_QCD_37x79_566 | 98.82 | XIa | 15 | 4332988 | 4092698 | 1 |
| Clostridium_difficile_QCD_63q42_QCD_63q42_563 | 98.03 | XIa | 28 | 4443737 | 4129288 | 1 |
| Clostridium_difficile_QCD_76w55_QCD_76w55_564 | 99.21 | XIa | 24 | 4396895 | 4078976 | 1 |
| Clostridium_difficile_QCD_97b34_QCD_97b34_565 | 99.21 | XIa | 15 | 4063610 | 3998408 | 1 |
| Clostridium_difficile_RA09_70_806 | 98.82 | XIa | 116 | 4232226 | 105769 | 13 |
| Clostridium_difficile_SA10_050_807 | 98.43 | XIa | 81 | 4319873 | 128043 | 11 |
| Clostridium_difficile_SG12_P1_791 | 98.43 | XIa | 102 | 4268551 | 146259 | 11 |
| Clostridium_difficile_T20_762 | 99.21 | XIa | 210 | 3815975 | 38353 | 30 |
| Clostridium_difficile_T22_771 | 98.82 | XIa | 332 | 4083325 | 32040 | 43 |
| Clostridium_difficile_T23_770 | 98.82 | XIa | 293 | 4078839 | 30654 | 37 |
| Clostridium_difficile_T3_776 | 98.82 | XIa | 275 | 4060583 | 34614 | 37 |
| Clostridium_difficile_T61_787 | 98.82 | XIa | 82 | 4071443 | 141610 | 9 |
| Clostridium_difficile_VL_0042_1181 | 98.82 | XIa | 377 | 4132263 | 66649 | 21 |
| Clostridium_difficile_VL_0048_974 | 98.03 | XIa | 245 | 4175645 | 199899 | 8 |
| Clostridium_difficile_VL_0052_907 | 98.43 | XIa | 214 | 4208030 | 161786 | 9 |
| Clostridium_difficile_VL_0059_1016 | 98.82 | XIa | 322 | 3880153 | 67831 | 18 |
| Clostridium_difficile_VL_0088_931 | 98.03 | XIa | 317 | 4456714 | 134792 | 10 |
| Clostridium_difficile_VL_0094_873 | 98.82 | XIa | 299 | 4119551 | 67547 | 18 |
| Clostridium_difficile_VL_0095_989 | 98.43 | XIa | 261 | 4197961 | 142480 | 9 |
| Clostridium_difficile_VL_0104_1105 | 98.03 | XIa | 261 | 4068388 | 88166 | 14 |
| Clostridium_difficile_VL_0108_993 | 98.82 | XIa | 322 | 4117060 | 106650 | 14 |
| Clostridium_difficile_VL_0114_995 | 98.82 | XIa | 339 | 4114836 | 59007 | 23 |
| Clostridium_difficile_VL_0119_998 | 98.82 | XIa | 340 | 4124686 | 83417 | 18 |
| Clostridium_difficile_VL_0123_1000 | 98.43 | XIa | 267 | 4394265 | 65991 | 20 |
| Clostridium_difficile_VL_0135_917 | 98.43 | XIa | 218 | 4282195 | 202085 | 8 |
| Clostridium_difficile_VL_0174_1012 | 98.82 | XIa | 266 | 4156515 | 134544 | 9 |
| Clostridium_difficile_VL_0177_947 | 98.82 | XIa | 306 | 4124242 | 95020 | 16 |
| Clostridium_difficile_VL_0199_1035 | 98.82 | XIa | 266 | 4120144 | 108096 | 13 |
| Clostridium_difficile_VL_0232_1051 | 98.43 | XIa | 213 | 4123326 | 166166 | 10 |
| Clostridium_difficile_VL_0245_1065 | 98.43 | XIa | 315 | 4176184 | 90332 | 15 |
| Clostridium_difficile_VL_0259_941 | 98.43 | XIa | 376 | 4098661 | 52866 | 22 |
| Clostridium_difficile_VL_0291_896 | 98.82 | XIa | 278 | 3881849 | 90247 | 16 |
| Clostridium_difficile_VL_0305_1080 | 98.82 | XIa | 310 | 4159239 | 149863 | 8 |
| Clostridium_difficile_VL_0307_1081 | 98.43 | XIa | 255 | 4123594 | 195946 | 6 |
| Clostridium_difficile_VL_0308_1082 | 98.82 | XIa | 280 | 4239945 | 71640 | 18 |
| Clostridium_difficile_VL_0311_884 | 98.43 | XIa | 388 | 4254985 | 44720 | 29 |
| Clostridium_difficile_VL_0359_1101 | 98.82 | XIa | 215 | 4095701 | 58622 | 19 |
| Clostridium_difficile_VL_0404_1130 | 98.43 | XIa | 233 | 4088651 | 219919 | 7 |
| Clostridium_difficile_VL_0414_1134 | 99.21 | XIa | 369 | 4124988 | 56163 | 22 |
| Clostridium_difficile_VL_0417_1149 | 98.82 | XIa | 343 | 4130169 | 129218 | 10 |
| Clostridium_difficile_VL_0426_1188 | 98.82 | XIa | 370 | 4204351 | 70763 | 15 |
| Clostridium_difficile_VL_0429_891 | 98.43 | XIa | 323 | 4113210 | 46838 | 25 |
| Clostridium_difficile_VL_0452_1104 | 98.43 | XIa | 293 | 4408841 | 135522 | 12 |
| Clostridium_difficile_VL_0459_944 | 98.03 | XIa | 344 | 4382319 | 65636 | 21 |
| Clostridium_difficile_VL_0460_1139 | 98.82 | XIa | 325 | 4130158 | 114096 | 11 |
| Clostridium_difficile_VRECD0001_1264 | 98.82 | XIa | 48 | 4255504 | 799464 | 2 |
| Clostridium_difficile_VRECD0003_1255 | 98.82 | XIa | 52 | 4105252 | 219704 | 5 |
| Clostridium_difficile_VRECD0004_1262 | 98.82 | XIa | 25 | 4036206 | 652602 | 3 |
| Clostridium_difficile_VRECD0005_1265 | 98.43 | XIa | 27 | 4256197 | 675134 | 3 |
| Clostridium_difficile_VRECD0006_1268 | 98.43 | XIa | 28 | 4203969 | 598150 | 3 |
| Clostridium_difficile_VRECD0007_1267 | 98.82 | XIa | 52 | 4569562 | 635337 | 3 |
| Clostridium_difficile_VRECD0008_1263 | 98.82 | XIa | 32 | 4093125 | 414552 | 3 |
| Clostridium_difficile_VRECD0009_1257 | 98.82 | XIa | 54 | 4317067 | 473305 | 3 |
| Clostridium_difficile_VRECD0010_1233 | 98.82 | XIa | 27 | 4130133 | 592811 | 3 |
| Clostridium_difficile_VRECD0012_1258 | 98.82 | XIa | 22 | 4126592 | 678324 | 3 |
| Clostridium_difficile_VRECD0014_1260 | 98.82 | XIa | 26 | 4070644 | 567836 | 3 |
| Clostridium_difficile_VRECD0016_1261 | 98.82 | XIa | 31 | 4241280 | 347042 | 4 |
| Clostridium_difficile_VRECD0017_1350 | 98.43 | XIa | 27 | 4149640 | 535845 | 3 |
| Clostridium_difficile_VRECD0019_1344 | 98.43 | XIa | 27 | 4146662 | 306509 | 4 |
| Clostridium_difficile_VRECD0020_1346 | 98.43 | XIa | 33 | 4240856 | 498190 | 3 |
| Clostridium_difficile_VRECD0021_1345 | 98.82 | XIa | 25 | 4011448 | 613070 | 3 |
| Clostridium_difficile_VRECD0023_1347 | 98.43 | XIa | 47 | 4593015 | 301073 | 3 |
| Clostridium_difficile_VRECD0024_1349 | 98.43 | XIa | 26 | 4140268 | 599831 | 3 |
| Clostridium_difficile_VRECD0025_1357 | 98.43 | XIa | 27 | 4161988 | 686847 | 2 |
| Clostridium_difficile_VRECD0026_1348 | 98.43 | XIa | 32 | 4421543 | 599187 | 3 |
| Clostridium_difficile_VRECD0027_1355 | 98.43 | XIa | 30 | 4297925 | 380836 | 5 |
| Clostridium_difficile_VRECD0030_1359 | 98.43 | XIa | 28 | 4512569 | 543500 | 3 |
| Clostridium_difficile_VRECD0033_1361 | 98.82 | XIa | 29 | 4523828 | 593675 | 2 |
| Clostridium_difficile_VRECD0034_1351 | 98.43 | XIa | 27 | 4084869 | 402072 | 3 |
| Clostridium_difficile_VRECD0035_1354 | 98.43 | XIa | 40 | 4233861 | 556145 | 3 |
| Clostridium_difficile_VRECD0036_1360 | 98.82 | XIa | 31 | 4079714 | 588526 | 3 |
| Clostridium_difficile_VRECD0038_1271 | 98.43 | XIa | 23 | 4121922 | 683887 | 2 |
| Clostridium_difficile_VRECD0039_1237 | 98.82 | XIa | 39 | 4249962 | 321186 | 3 |
| Clostridium_difficile_VRECD0041_1273 | 98.43 | XIa | 39 | 4357659 | 421412 | 4 |
| Clostridium_difficile_VRECD0042_1274 | 98.43 | XIa | 38 | 4169930 | 385482 | 4 |
| Clostridium_difficile_VRECD0043_1206 | 98.43 | XIa | 27 | 4139048 | 539782 | 2 |
| Clostridium_difficile_VRECD0047_1276 | 98.43 | XIa | 29 | 4154535 | 475562 | 4 |
| Clostridium_difficile_VRECD0048_1277 | 98.82 | XIa | 26 | 4065912 | 562478 | 3 |
| Clostridium_difficile_VRECD0049_1278 | 98.82 | XIa | 36 | 4205910 | 579078 | 3 |
| Clostridium_difficile_VRECD0051_1279 | 98.43 | XIa | 42 | 4198487 | 402206 | 4 |
| Clostridium_difficile_VRECD0053_1281 | 98.82 | XIa | 30 | 4308716 | 560877 | 2 |
| Clostridium_difficile_VRECD0054_1284 | 98.82 | XIa | 49 | 4209694 | 402151 | 4 |
| Clostridium_difficile_VRECD0055_1283 | 98.43 | XIa | 30 | 4196308 | 693711 | 2 |
| Clostridium_difficile_VRECD0057_1363 | 98.43 | XIa | 23 | 4121104 | 580367 | 3 |
| Clostridium_difficile_VRECD0058_1364 | 98.82 | XIa | 30 | 4191407 | 497568 | 3 |
| Clostridium_difficile_VRECD0059_1369 | 98.82 | XIa | 32 | 4187721 | 562268 | 3 |
| Clostridium_difficile_VRECD0060_1365 | 98.82 | XIa | 36 | 4189636 | 314543 | 5 |
| Clostridium_difficile_VRECD0061_1370 | 98.82 | XIa | 29 | 4121024 | 542064 | 4 |
| Clostridium_difficile_VRECD0063_1322 | 98.82 | XIa | 30 | 4185963 | 561804 | 3 |
| Clostridium_difficile_VRECD0064_1372 | 98.82 | XIa | 26 | 4184952 | 561836 | 3 |
| Clostridium_difficile_VRECD0065_1373 | 98.82 | XIa | 27 | 4187935 | 562286 | 3 |
| Clostridium_difficile_VRECD0067_1367 | 98.82 | XIa | 33 | 4194047 | 687659 | 3 |
| Clostridium_difficile_VRECD0070_1374 | 98.43 | XIa | 36 | 4239257 | 311198 | 4 |
| Clostridium_difficile_VRECD0071_1375 | 98.43 | XIa | 42 | 4237804 | 511935 | 3 |
| Clostridium_difficile_VRECD0072_1376 | 98.82 | XIa | 30 | 4186963 | 562426 | 3 |
| Clostridium_difficile_VRECD0073_1286 | 98.82 | XIa | 33 | 4320443 | 568445 | 3 |
| Clostridium_difficile_VRECD0078_1243 | 98.82 | XIa | 45 | 3981831 | 187468 | 7 |
| Clostridium_difficile_VRECD0082_1250 | 98.43 | XIa | 41 | 4308862 | 239429 | 5 |
| Clostridium_difficile_VRECD0083_1291 | 98.43 | XIa | 43 | 4387145 | 262957 | 5 |
| Clostridium_difficile_VRECD0084_1293 | 98.82 | XIa | 46 | 4389239 | 500411 | 3 |
| Clostridium_difficile_VRECD0085_1251 | 98.82 | XIa | 30 | 4074943 | 380392 | 4 |
| Clostridium_difficile_VRECD0089_1324 | 98.82 | XIa | 33 | 4114466 | 296534 | 6 |
| Clostridium_difficile_VRECD0095_1326 | 98.82 | XIa | 28 | 4174911 | 257511 | 6 |
| Clostridium_difficile_VRECD0099_1295 | 98.82 | XIa | 29 | 4165342 | 483795 | 4 |
| Clostridium_difficile_VRECD0100_1294 | 98.43 | XIa | 34 | 4419253 | 600146 | 3 |
| Clostridium_difficile_VRECD0101_1296 | 98.82 | XIa | 28 | 4274243 | 485062 | 4 |
| Clostridium_difficile_VRECD0102_1297 | 98.82 | XIa | 31 | 4098928 | 597153 | 3 |
| Clostridium_difficile_VRECD0103_1298 | 98.43 | XIa | 32 | 4199524 | 508865 | 3 |
| Clostridium_difficile_VRECD0106_1301 | 98.82 | XIa | 23 | 4045108 | 653419 | 2 |
| Clostridium_difficile_VRECD0109_1385 | 98.82 | XIa | 30 | 4098893 | 563074 | 3 |
| Clostridium_difficile_VRECD0110_1386 | 98.82 | XIa | 50 | 4238718 | 498953 | 3 |
| Clostridium_difficile_VRECD0112_1302 | 98.82 | XIa | 40 | 4266439 | 561758 | 3 |
| Clostridium_difficile_VRECD0115_1305 | 98.82 | XIa | 28 | 4096161 | 546233 | 3 |
| Clostridium_difficile_VRECD0116_1327 | 98.43 | XIa | 97 | 4571183 | 167119 | 11 |
| Clostridium_difficile_VRECD0119_1229 | 98.82 | XIa | 41 | 4347268 | 789394 | 3 |
| Clostridium_difficile_VRECD0122_1252 | 98.82 | XIa | 47 | 4114017 | 179488 | 7 |
| Clostridium_difficile_VRECD0123_1223 | 99.21 | XIa | 38 | 3945589 | 202201 | 5 |
| Clostridium_difficile_VRECD0127_1230 | 98.43 | XIa | 40 | 4431161 | 707933 | 3 |
| Clostridium_difficile_VRECD0128_1238 | 98.82 | XIa | 357 | 4448160 | 598450 | 3 |
| Clostridium_difficile_VRECD0138_1213 | 98.43 | XIa | 37 | 4291619 | 362902 | 5 |
| Clostridium_difficile_VRECD0139_1236 | 98.82 | XIa | 35 | 4446093 | 682760 | 3 |
| Clostridium_difficile_VRECD0140_1224 | 98.43 | XIa | 40 | 4282826 | 368740 | 5 |
| Clostridium_difficile_VRECD0141_1211 | 98.43 | XIa | 33 | 4291501 | 450759 | 4 |
| Clostridium_difficile_VRECD0144_1309 | 98.43 | XIa | 35 | 4120089 | 402297 | 4 |
| Clostridium_difficile_VRECD0145_1310 | 98.82 | XIa | 40 | 4210086 | 698250 | 2 |
| Clostridium_difficile_VRECD0146_1311 | 98.43 | XIa | 36 | 4294414 | 526365 | 4 |
| Clostridium_difficile_VRECD0148_1313 | 98.43 | XIa | 65 | 4252825 | 160557 | 10 |
| Clostridium_difficile_VRECD0149_1314 | 98.43 | XIa | 39 | 4116743 | 221331 | 7 |
| Clostridium_difficile_VRECD0153_1330 | 98.43 | XIa | 55 | 4225485 | 343945 | 4 |
| Clostridium_difficile_VRECD0154_1318 | 98.82 | XIa | 38 | 4147832 | 414616 | 3 |
| Clostridium_difficile_VRECD0157_1321 | 98.03 | XIa | 54 | 4397606 | 268671 | 6 |
| Clostridium_difficile_VRECD0158_1329 | 98.03 | XIa | 56 | 4396137 | 205249 | 8 |
| Clostridium_difficile_VRECD0159_1216 | 98.43 | XIa | 34 | 4316578 | 394724 | 4 |
| Clostridium_difficile_VRECD0160_1221 | 98.43 | XIa | 29 | 4178309 | 529907 | 4 |
| Clostridium_difficile_VRECD0162_1226 | 98.82 | XIa | 31 | 4062673 | 308123 | 5 |
| Clostridium_difficile_VRECD0163_1219 | 98.43 | XIa | 37 | 4231218 | 346917 | 4 |
| Clostridium_difficile_VRECD0165_1212 | 98.43 | XIa | 44 | 4248821 | 422442 | 3 |
| Clostridium_difficile_VRECD0166_1249 | 98.43 | XIa | 33 | 4168589 | 505747 | 3 |
| Clostridium_difficile_VRECD0167_1234 | 98.82 | XIa | 55 | 4289170 | 501840 | 4 |
| Clostridium_difficile_VRECD0169_1245 | 98.43 | XIa | 30 | 4057998 | 387103 | 4 |
| Clostridium_difficile_VRECD0170_1244 | 98.43 | XIa | 39 | 4222769 | 477083 | 4 |
| Clostridium_difficile_VRECD0171_1253 | 98.82 | XIa | 38 | 4325883 | 263908 | 7 |
| Clostridium_difficile_VRECD0175_1235 | 98.82 | XIa | 26 | 4366076 | 632922 | 3 |
| Clostridium_difficile_VRECD0177_1222 | 98.82 | XIa | 28 | 4282513 | 454161 | 3 |
| Clostridium_difficile_VRECD0178_1241 | 98.82 | XIa | 37 | 4257551 | 571332 | 4 |
| Clostridium_difficile_VRECD0179_1256 | 98.43 | XIa | 34 | 4338827 | 428504 | 4 |
| Clostridium_difficile_VRECD0180_1332 | 98.82 | XIa | 43 | 4290419 | 222418 | 7 |
| Clostridium_difficile_VRECD0182_1334 | 98.43 | XIa | 37 | 4294031 | 242766 | 7 |
| Clostridium_difficile_VRECD0183_1335 | 98.43 | XIa | 38 | 4173270 | 519461 | 3 |
| Clostridium_difficile_VRECD0184_1336 | 98.82 | XIa | 33 | 4110442 | 512879 | 3 |
| Clostridium_difficile_VRECD0185_1337 | 98.43 | XIa | 30 | 4250524 | 341755 | 4 |
| Clostridium_difficile_VRECD0189_1339 | 98.03 | XIa | 45 | 4245033 | 193494 | 7 |
| Clostridium_difficile_VRECD0190_1338 | 98.43 | XIa | 30 | 4169585 | 271547 | 5 |
| Clostridium_difficile_VRECD0191_1340 | 98.43 | XIa | 48 | 4172317 | 184561 | 9 |
| Clostridium_difficile_Y10_682 | 98.43 | XIa | 37 | 4062917 | 237821 | 6 |
| Clostridium_difficile_Y155_685 | 98.03 | XIa | 232 | 3980239 | 33044 | 36 |
| Clostridium_difficile_Y165_704 | 85.04 | XIa | 367 | 4263767 | 24471 | 55 |
| Clostridium_difficile_Y231_707 | 98.82 | XIa | 42 | 4093690 | 197543 | 8 |
| Clostridium_difficile_Y247_694 | 97.64 | XIa | 44 | 4053085 | 199386 | 7 |
| Clostridium_difficile_Y270_705 | 98.82 | XIa | 53 | 4149527 | 217760 | 8 |
| Clostridium_difficile_Y312_706 | 98.82 | XIa | 160 | 4054373 | 47451 | 26 |
| Clostridium_difficile_Y381_713 | 98.82 | XIa | 60 | 4060729 | 234466 | 8 |
| Clostridium_difficile_Y401_686 | 98.43 | XIa | 205 | 4000151 | 35327 | 36 |
| Clostridium_difficile_Y41_684 | 98.03 | XIa | 39 | 4074379 | 233399 | 7 |
| Clostridium_sp._HMC19D07_459 | 98.43 | Xia | 98 | 4217734 | 139497 | 12 |
| Clostridium_sp._HMSC19A11_449 | 98.82 | Xia | 117 | 4235516 | 136274 | 9 |
| Clostridium_sp._HMSC19B01_448 | 99.21 | Xia | 101 | 4083546 | 118969 | 12 |
| Clostridium_sp._HMSC19B04_451 | 98.43 | Xia | 68 | 3979326 | 184637 | 9 |
| Clostridium_sp._HMSC19B10_450 | 99.21 | Xia | 77 | 4056622 | 128616 | 11 |
| Clostridium_sp._HMSC19B11_452 | 98.82 | Xia | 136 | 4137486 | 91682 | 15 |
| Clostridium_sp._HMSC19B12_453 | 98.43 | Xia | 113 | 4286468 | 138393 | 11 |
| Clostridium_sp._HMSC19C05_454 | 99.21 | Xia | 69 | 4062786 | 118779 | 12 |
| Clostridium_sp._HMSC19C08_455 | 98.82 | Xia | 79 | 4069234 | 128619 | 11 |
| Clostridium_sp._HMSC19C09_456 | 99.21 | Xia | 91 | 4060413 | 113889 | 14 |
| Clostridium_sp._HMSC19C11_457 | 98.82 | Xia | 79 | 4015638 | 152320 | 10 |
| Clostridium_sp._HMSC19D02_458 | 98.82 | Xia | 85 | 4070187 | 157295 | 9 |
| Clostridium_sp._HMSC19E03_460 | 99.21 | Xia | 64 | 4059828 | 134413 | 10 |
| Clostridium_aerotolerans_DSM_5434_165 | 98.43 | XIVa | 50 | 4732373 | 213027 | 7 |
| Clostridium_aminophilum_542 | 98.82 | XIVa | 31 | 3113820 | 176596 | 6 |
| Clostridium_aminophilum_DSM_10710_178 | 98.82 | XIVa | 36 | 3114416 | 176585 | 6 |
| Clostridium_aminophilum_KH1P1_540 | 98.43 | XIVa | 108 | 3198475 | 54860 | 19 |
| Clostridium_bolteae_90A5_110 | 98.03 | XIVa | 12 | 6421395 | 1978325 | 2 |
| Clostridium_bolteae_90A9_109 | 98.43 | XIVa | 1 | 6377378 | 6377378 | 1 |
| Clostridium_bolteae_90B3_108 | 98.03 | XIVa | 4 | 6538460 | 3794250 | 1 |
| Clostridium_bolteae_90B7_107 | 98.03 | XIVa | 19 | 6439235 | 799384 | 4 |
| Clostridium_bolteae_90B8_106 | 98.43 | XIVa | 21 | 6482686 | 663201 | 5 |
| Clostridium_bolteae_ATCC_BAA_613_26 | 97.64 | XIVa | 68 | 6557988 | 187711 | 12 |
| Clostridium_bolteae_WAL_14578_291 | 98.03 | XIVa | 30 | 6604884 | 668224 | 3 |
| Clostridium_celerecrescens_152B_206 | 97.64 | XIVa | 92 | 5038011 | 208566 | 9 |
| Clostridium_citroniae_WAL_17108_63 | 98.03 | XIVa | 41 | 6647380 | 473033 | 3 |
| Clostridium_citroniae_WAL19142_292 | 98.03 | XIVa | 38 | 6252818 | 694783 | 4 |
| Clostridium_clostridioforme_2_1_49FAA_65 | 98.43 | XIVa | 69 | 5500475 | 722831 | 3 |
| Clostridium_clostridioforme_2789STDY5834865_334 | 98.43 | XIVa | 202 | 5514222 | 70965 | 22 |
| Clostridium_clostridioforme_90A1_104 | 98.43 | XIVa | 16 | 5806027 | 1108021 | 2 |
| Clostridium_clostridioforme_90A3_103 | 98.43 | XIVa | 11 | 5549890 | 1495260 | 2 |
| Clostridium_clostridioforme_90A4_102 | 98.82 | XIVa | 48 | 5871489 | 217308 | 10 |
| Clostridium_clostridioforme_90A6_101 | 98.43 | XIVa | 22 | 6033914 | 582564 | 3 |
| Clostridium_clostridioforme_90A7_100 | 98.43 | XIVa | 8 | 6180373 | 1761752 | 2 |
| Clostridium_clostridioforme_90A8_99 | 98.03 | XIVa | 47 | 5974284 | 254783 | 9 |
| Clostridium_clostridioforme_90B1_98 | 98.03 | XIVa | 10 | 5602152 | 1271686 | 2 |
| Clostridium_clostridioforme_AGR2157_122 | 98.43 | XIVa | 133 | 4943165 | 83167 | 20 |
| Clostridium_clostridioforme_ATCC_25537_544 | 98.03 | XIVa | 148 | 5465751 | 80688 | 20 |
| Clostridium_clostridioforme_CM201_95 | 98.03 | XIVa | 7 | 5655915 | 1153171 | 2 |
| Clostridium_clostridioforme_NLAE_zl_C196_539 | 98.43 | XIVa | 164 | 5225716 | 59867 | 27 |
| Clostridium_clostridioforme_NLAE_zl_G208_41 | 98.82 | XIVa | 157 | 5236575 | 65106 | 24 |
| Clostridium_clostridioforme_WAL_7855_293 | 98.43 | XIVa | 70 | 5459495 | 262765 | 5 |
| Clostridium_glycyrrhizinilyticum_JCM_13369_328 | 94.49 | XIVa | 65 | 3215420 | 112462 | 10 |
| Clostridium_hylemonae_DSM_15053_32 | 98.43 | XIVa | 123 | 3889859 | 2898417 | 1 |
| Clostridium_ihumii_AP5_155 | 99.21 | XIVa | 96 | 4433668 | 124325 | 10 |
| Clostridium_indolis_DSM_755_145 | 98.82 | XIVa | 1 | 6383701 | 6383701 | 1 |
| Clostridium_methoxybenzovorans_SR3_117 | 98.82 | XIVa | 14 | 7085377 | 1607802 | 2 |
| Clostridium_methylpentosum_DSM_5476_35 | 98.43 | XIVa | 17 | 3478423 | 779329 | 2 |
| Clostridium_saccharolyticum_WM1_22 | 98.82 | XIVa | 1 | 4662871 | 4662871 | 1 |
| Clostridium_sp._12A_144 | 98.82 | XIVa | 3 | 4605255 | 3532345 | 1 |
| Clostridium_sp._7_3_54FAA_64 | 98.43 | XIVa | 51 | 5464886 | 459666 | 5 |
| Clostridium_sp._ASBs410_154 | 99.21 | XIVa | 1 | 5723672 | 5723672 | 1 |
| Clostridium_sp._ATCC_29733_130 | 90.55 | XIVa | 161 | 3084735 | 45185 | 19 |
| Clostridium_sp._BR31_446 | 99.61 | XIVa | 64 | 3318223 | 154838 | 7 |
| Clostridium_sp._DSM_4029_550 | 91.73 | XIVa | 11 | 3113497 | 984592 | 2 |
| Clostridium_sp._FS41_257 | 98.03 | XIVa | 97 | 6265866 | 227665 | 9 |
| Clostridium_sp._M62_1_36 | 97.64 | XIVa | 26 | 3842594 | 463016 | 3 |
| Clostridium_sp._Marseille_P2415_560 | 98.43 | XIVa | 9 | 5247868 | 4178102 | 1 |
| Clostridium_sp._Marseille_P2538_535 | 99.21 | XIVa | 2 | 4144149 | 4143223 | 1 |
| Clostridium_sp._Marseille_P3244_547 | 98.43 | XIVa | 3 | 2972275 | 2607599 | 1 |
| Clostridium_sp._SY8519_73 | 98.82 | XIVa | 1 | 2835737 | 2835737 | 1 |
| Clostridium_symbiosum_2789STDY5834864_339 | 98.82 | XIVa | 186 | 4727130 | 102800 | 13 |
| Clostridium_symbiosum_ATCC_14940_129 | 98.43 | XIVa | 270 | 4823675 | 30142 | 49 |
| Clostridium_symbiosum_WAL_14163_54 | 98.82 | XIVa | 52 | 5352498 | 328026 | 6 |
| Clostridium_symbiosum_WAL_14673_55 | 98.82 | XIVa | 55 | 4916964 | 628413 | 4 |
| Clostridium_lactatifermentans_DSM_14214_559 | 98.82 | XIVb | 163 | 3546077 | 42744 | 26 |
| Clostridium_neopropionicum_DSM_3847_363 | 98.03 | XIVb | 29 | 3194881 | 311201 | 4 |
| Clostridium_propionicum_DSM_1682_548 | 97.64 | XIVb | 28 | 3076693 | 170419 | 5 |
| Clostridium_propionicum_DSM_1682_X2_362 | 96.85 | XIVb | 1 | 3120417 | 3120417 | 1 |
| Clostridium_sp._ASF356_93 | 98.82 | XIVb | 6 | 2912727 | 1039841 | 2 |
| Clostridium_difficile_P28_690_innocuum | 87.8 | XVI | 81 | 4323423 | 92019 | 15 |
| Clostridium_innocuum_2789STDY5834853_341 | 87.01 | XVI | 45 | 4283273 | 225726 | 7 |
| Clostridium_innocuum_2959_96 | 86.61 | XVI | 7 | 4803668 | 1547824 | 2 |
| Clostridium_innocuum_NLAE_zl_C381_546 | 87.01 | XVI | 56 | 4232065 | 181667 | 9 |

^1^ Genome completness as measured by identification of Benchmarking Universal Single Copy Orthologs

^2^ Clostridial cluster: Cluster number from the Clostridial phylogenetic reconstruction shown in Figure 1.

^3^ N50: length such that sequence contigs of this length or longer include half the bases of the assembly.

^4^ L50: number of sequence contigs that are longer than, or equal to, the N50 length and therefore include half the bases of the assembly.

**Table S2.** **Conserved proteins found among the genomes listed in Table S1**. The sequences of these protein families were used to construct the species tree shown in Figure 1, best amino acid substitution models calculated for each partition in the correspondent matrix are listed.

| **Protein Family** | **Annotation** | **Length^1^** | **Start^2^** | **End^2^** | **Model^3^** |
| --- | --- | --- | --- | --- | --- |
| 1 | Pyruvate:ferredoxin (flavodoxin) oxidoreductase | 1181 | 1 | 1181 | LG+F+I+G4 |
| 2 | Valine--tRNA ligase | 885 | 1182 | 2066 | LG+F+I+G4 |
| 3 | Polyribonucleotide nucleotidyltransferase | 707 | 2067 | 2773 | LG+F+I+G4 |
| 4 | Putative RNA-binding protein | 716 | 2774 | 3489 | LG+F+I+G4 |
| 5 | Aspartate-tRNA ligase | 590 | 3488 | 4079 | LG+I+G4 |
| 6 | Methionine-tRNA ligase | 646 | 4080 | 4725 | LG+F+I+G4 |
| 7 | Excinuclease ABC subunit UvrB | 656 | 4726 | 5381 | LG+F+I+G4 |
| 8 | tRNA uridine-5-carboxymethylaminomethyl(34) synthesis enzyme MnmG | 626 | 5382 | 6007 | LG+F+I+G4 |
| 9 | Elongation factor 4 | 600 | 6008 | 6607 | LG+F+I+G4 |
| 10 | Molecular chaperone DnaK | 616 | 6608 | 7223 | LG+F+I+G4 |
| 11 | Formate-tetrahydrofolate ligase | 557 | 7224 | 7780 | LG+I+G4 |
| 12 | Asparagine--tRNA ligase | 463 | 7781 | 8243 | LG+F+I+G4 |
| 13 | Nicotinate phosphoribosyltransferase | 474 | 8244 | 8717 | LG+F+I+G4 |
| 14 | Chaperonin GroEL | 542 | 8718 | 9259 | LG+F+I+G4 |
| 15 | Ribonuclease Y | 520 | 9260 | 9779 | LG+I+G4 |
| 16 | Methionine adenosyltransferase | 389 | 9780 | 10168 | LG+F+I+G4 |
| 17 | Recombinase RecA | 345 | 10169 | 10513 | LG+F+I+G4 |
| 18 | Flavodoxin-dependent (E)-4-hydroxy-3-methylbut-2-enyl-diphosphate synthase | 350 | 10514 | 10863 | LG+F+I+G4 |
| 19 | 4-hydroxy-tetrahydrodipicolinate synthase | 296 | 10864 | 11159 | LG+I+G4 |
| 20 | Peptide chain release factor 1 | 356 | 11160 | 11515 | LG+I+G4 |
| 21 | Holliday junction branch migration DNA helicase RuvB | 334 | 11516 | 11849 | LG+I+G4 |
| 22 | 6-phosphofructokinase | 319 | 11850 | 12168 | LG+I+G4 |
| 23 | Uracil phosphoribosyltransferase | 209 | 12169 | 12377 | LG+I+G4 |
| 24 | ATP-dependent Clp endopeptidase, proteolytic subunit ClpP | 194 | 12378 | 12571 | LG+I+G4 |
| 25 | 30S ribosomal protein S15 | 86 | 12572 | 12657 | LG+F+I+G4 |
| 26 | 50S ribosomal protein L21 | 103 | 12658 | 12760 | LG+I+G4 |
| 27 | 30S ribosomal protein S16 | 76 | 12761 | 12836 | LG+I+G4 |

**^1^** Length of the sequences corresponding to each protein family included in the concatenated sequence alignment (matrix) used for the species tree shown in Figure 1.

**^2^** Location of the sequences belonging to each protein family within the concatenated sequence alignment (matrix), i. e. coordinates of the partitions.

**^3^** Best amino acid exchange rate model for each protein family sequence alignment (partition).

**Table S3.** **Conserved proteins families found among the 179 genomes with N50>600 kbp listed in Table S1**. The sequences of these protein families were used to construct the species tree shown in Supplementary Figure 16, best amino acid substitution models calculated for each partition in the correspondent matrix are listed.

| **Protein family** | **Annotation** | **Length^1^** | **Start^2^** | **End^2^** | **Model^3^** |
| --- | --- | --- | --- | --- | --- |
| 1 | DNA-directed RNA polymerase beta subunit (EC 2.7.7.6) | 1196 | 1 | 1196 | LG+I+G4 |
| 2 | DNA-directed RNA polymerase beta' subunit (EC 2.7.7.6) | 1134 | 1197 | 2330 | LG+I+G4 |
| 3 | Pyruvate-flavodoxin oxidoreductase (EC 1.2.7.-) | 1188 | 2331 | 3518 | LG+F+I+G4 |
| 4 | Excinuclease ABC subunit A | 533 | 3519 | 4051 | LG+F+I+G4 |
| 5 | Valyl-tRNA synthetase (EC 6.1.1.9) | 523 | 4052 | 4574 | LG+F+I+G4 |
| 6 | Alanyl-tRNA synthetase (EC 6.1.1.7) | 106 | 4575 | 4680 | WAG+I+G4 |
| 7 | ATP-dependent Clp protease, ATP-binding subunit ClpC | 799 | 4681 | 5479 | LG+F+I+G4 |
| 8 | DNA gyrase subunit A (EC 5.99.1.3) | 817 | 5480 | 6296 | LG+F+I+G4 |
| 9 | Transcription accessory protein (S1 RNA-binding domain) | 469 | 6297 | 6765 | LG+F+I+G4 |
| 10 | Translation elongation factor G | 686 | 6766 | 7451 | LG+F+I+G4 |
| 11 | Excinuclease ABC subunit B | 396 | 7452 | 7847 | LG+F+I+G4 |
| 12 | tRNA uridine 5-carboxymethylaminomethyl modification enzyme GidA | 619 | 7848 | 8466 | LG+F+I+G4 |
| 13 | Lysyl-tRNA synthetase (class II) (EC 6.1.1.6) | 497 | 8467 | 8963 | LG+I+G4 |
| 14 | Phosphoglycerate kinase (EC 2.7.2.3) | 390 | 8964 | 9353 | LG+F+I+G4 |
| 15 | Aspartyl-tRNA synthetase (EC 6.1.1.12) | 563 | 9354 | 9916 | LG+I+G4 |
| 16 | Chaperone protein DnaK | 605 | 9917 | 10521 | LG+F+I+G4 |
| 17 | Translation elongation factor LepA | 371 | 10522 | 10892 | LG+I+G4 |
| 18 | Formate--tetrahydrofolate ligase (EC 6.3.4.3) | 473 | 10893 | 11365 | LG+F+I+G4 |
| 19 | Heat shock protein 60 family chaperone GroEL | 526 | 11366 | 11891 | LG+F+I+G4 |
| 20 | GMP synthase [glutamine-hydrolyzing], amidotransferase subunit (EC 6.3.5.2) | 508 | 11892 | 12399 | LG+F+I+G4 |
| 21 | Ribonuclease Y | 478 | 12400 | 12877 | LG+I+G4 |
| 22 | Asparaginyl-tRNA synthetase (EC 6.1.1.22) | 414 | 12878 | 13291 | LG+I+G4 |
| 23 | Glucose-6-phosphate isomerase (EC 5.3.1.9) | 446 | 13292 | 13737 | LG+F+I+G4 |
| 24 | GTP-binding protein EngA | 436 | 13738 | 14173 | LG+I+G4 |
| 25 | Enolase (EC 4.2.1.11) | 413 | 14174 | 14586 | LG+I+G4 |
| 26 | Signal recognition particle, subunit Ffh SRP54 (TC 3.A.5.1.1) | 435 | 14587 | 15021 | LG+F+I+G4 |
| 27 | RNA polymerase sigma factor RpoD | 333 | 15022 | 15354 | LG+I+G4 |
| 28 | ATP-dependent Clp protease ATP-binding subunit ClpX | 380 | 15355 | 15734 | LG+I+G4 |
| 29 | Acetate kinase (EC 2.7.2.1) | 385 | 15735 | 16119 | LG+F+I+G4 |
| 30 | Cysteine desulfurase (EC 2.8.1.7) | 377 | 16120 | 16496 | LG+F+I+G4 |
| 31 | S-adenosylmethionine synthetase (EC 2.5.1.6) | 389 | 16497 | 16885 | LG+I+G4 |
| 32 | RecA protein | 335 | 16886 | 17220 | LG+I+G4 |
| 33 | S-adenosylmethionine:tRNA ribosyltransferase-isomerase | 340 | 17221 | 17560 | LG+F+I+G4 |
| 34 | 1-hydroxy-2-methyl-2-(E)-butenyl 4-diphosphate synthase (EC 1.17.7.1) | 350 | 17561 | 17910 | LG+F+I+G4 |
| 35 | GTP-binding and nucleic acid-binding protein YchF | 365 | 17911 | 18275 | LG+I+G4 |
| 36 | Peptide chain release factor 2 | 308 | 18276 | 18583 | LG+I+G4 |
| 37 | Peptide chain release factor 1 | 354 | 18584 | 18937 | LG+I+G4 |
| 38 | TsaD/Kae1/Qri7 protein, required for threonylcarbamoyladenosine t(6)A37 formation in tRNA | 265 | 18938 | 19202 | LG+I+G4 |
| 39 | Holliday junction DNA helicase RuvB | 328 | 19203 | 19530 | LG+I+G4 |
| 40 | GTP-binding protein Era | 294 | 19531 | 19824 | LG+F+I+G4 |
| 41 | DNA-directed RNA polymerase alpha subunit (EC 2.7.7.6) | 315 | 19825 | 20139 | LG+I+G4 |
| 42 | Ribosomal large subunit pseudouridine synthase D (EC 4.2.1.70) | 301 | 20140 | 20440 | cpREV+I+G4 |
| 43 | rRNA small subunit methyltransferase H | 311 | 20441 | 20751 | LG+F+I+G4 |
| 44 | Signal peptidase-like protein | 264 | 20752 | 21015 | LG+I+G4 |
| 45 | Predicted P-loop-containing kinase | 283 | 21016 | 21298 | LG+I+G4 |
| 46 | LSU ribosomal protein L2p (L8e) | 261 | 21299 | 21559 | LG+I+G4 |
| 47 | Transmembrane component of general energizing module of ECF transporters | 259 | 21560 | 21818 | LG+F+I+G4 |
| 48 | SSU ribosomal protein S3p (S3e) | 213 | 21819 | 22031 | LG+I+G4 |
| 49 | SSU ribosomal protein S2p (SAe) | 202 | 22032 | 22233 | LG+I+G4 |
| 50 | Septum site-determining protein MinD | 244 | 22234 | 22477 | LG+I+G4 |
| 51 | RNA polymerase sporulation specific sigma factor SigE | 221 | 22478 | 22698 | LG+I+G4 |
| 52 | RNA polymerase sporulation specific sigma factor SigG | 256 | 22699 | 22954 | LG+G4 |
| 53 | LSU ribosomal protein L1p (L10Ae) | 229 | 22955 | 23183 | LG+I+G4 |
| 54 | Redox-sensitive transcriptional regulator (AT-rich DNA-binding protein) | 167 | 23184 | 23350 | LG+I+G4 |
| 55 | LSU ribosomal protein L3p (L3e) | 207 | 23351 | 23557 | LG+I+G4 |
| 56 | Uracil phosphoribosyltransferase (EC 2.4.2.9) | 209 | 23558 | 23766 | LG+G4 |
| 57 | LSU ribosomal protein L4p (L1e) | 169 | 23767 | 23935 | LG+I+G4 |
| 58 | SSU ribosomal protein S4p (S9e) | 101 | 23936 | 24036 | LG+I+G4 |
| 59 | ATP-dependent Clp protease proteolytic subunit (EC 3.4.21.92) | 192 | 24037 | 24228 | LG+I+G4 |
| 60 | Translation initiation factor 3 | 123 | 24229 | 24351 | LG+I+G4 |
| 61 | Translation elongation factor P | 185 | 24352 | 24536 | LG+G4 |
| 62 | Transcription antitermination protein NusG | 171 | 24537 | 24707 | LG+I+G4 |
| 63 | LSU ribosomal protein L6p (L9e) | 179 | 24708 | 24886 | LG+I+G4 |
| 64 | LSU ribosomal protein L5p (L11e) | 179 | 24887 | 25065 | LG+I+G4 |
| 65 | SSU ribosomal protein S7p (S5e) | 177 | 25066 | 25242 | LG+I+G4 |
| 66 | SSU ribosomal protein S5p (S2e) | 73 | 25243 | 25315 | LG+G4 |
| 67 | tmRNA-binding protein SmpB | 151 | 25316 | 25466 | LG+I+G4 |
| 68 | Transcription elongation factor GreA | 158 | 25467 | 25624 | LG+I+G4 |
| 69 | LSU ribosomal protein L15p (L27Ae) | 92 | 25625 | 25716 | LG+I+G4 |
| 70 | SSU ribosomal protein S8p (S15Ae) | 132 | 25717 | 25848 | LG+I+G4 |
| 71 | LSU ribosomal protein L14p (L23e) | 122 | 25849 | 25970 | LG+I+G4 |
| 72 | LSU ribosomal protein L20p | 117 | 25971 | 26087 | LG+G4 |
| 73 | LSU ribosomal protein L21p | 101 | 26088 | 26188 | LG+I+G4 |
| 74 | SSU ribosomal protein S15p (S13e) | 80 | 26189 | 26268 | LG+G4 |
| 75 | SSU ribosomal protein S17p (S11e) | 67 | 26269 | 26335 | FLU+I+G4 |
| 76 | SSU ribosomal protein S16p | 76 | 26336 | 26411 | LG+G4 |
| 77 | KH domain RNA binding protein YlqC | 50 | 26412 | 26461 | rtREV+G4 |
| 78 | Translation initiation factor 1 | 72 | 26462 | 26533 | LG+G4 |
| 79 | LSU ribosomal protein L11p (L12e) | 90 | 26534 | 26623 | LG+I+G4 |

**^1^** Length of the sequences corresponding to each protein family included in the concatenated sequence alignment (matrix) used for the species tree shown in Figure S16.

**^2^** Location of the sequences belonging to each protein family within the concatenated sequence alignment (matrix), i. e. coordinates of the partitions.

**^3^** Best amino acid exchange rate model for each protein family sequence alignment (partition).

**Table S4. Distribution of toxin homologues in *Clostridium* species. “**X” indicates that the species has the homolog of the toxin.

|  |  | **Toxins** | | | |
| --- | --- | --- | --- | --- | --- |
|  |  | *C. difficile*  A/B toxins* | *C. perfringens* alpha toxin | *C. septicum* alpha toxin | *C. botulinum/tetani*  toxin |
| Taxonomic group | *C. difficile* | X |  |  |  |
|  | *C. acetobutylicum* | X |  |  |  |
|  | *C. sordelli* | X | X |  |  |
|  | *C. novyi* | X | X | X |  |
|  | *C. haemolyticum* |  | X | X |  |
|  | *C. botulinum C & D* |  | X | X | X |
|  | *C. septicum* |  |  | X |  |
|  | *C. perfringens* |  | X |  |  |
|  | *C. cavendishii* |  | X |  |  |
|  | *C. dakarense* |  | X |  |  |
|  | *C. argentinense* |  | X |  | X |
|  | *C. baratii* |  | X |  | X |
|  | *C. tetani* |  |  |  | X |
|  | *C. butyricum* |  |  |  | X |
|  | *C. botulinum A, B, E & F* |  |  |  | X |

*****TpeL from *C. perfringens* has been previously defined as a homolog of toxins A and B from *C. difficile* (Amimoto et al., Microbiology, 2007, 153:1198-206), however, they are largely divergent in the used genomes and therefore not included in this analysis.


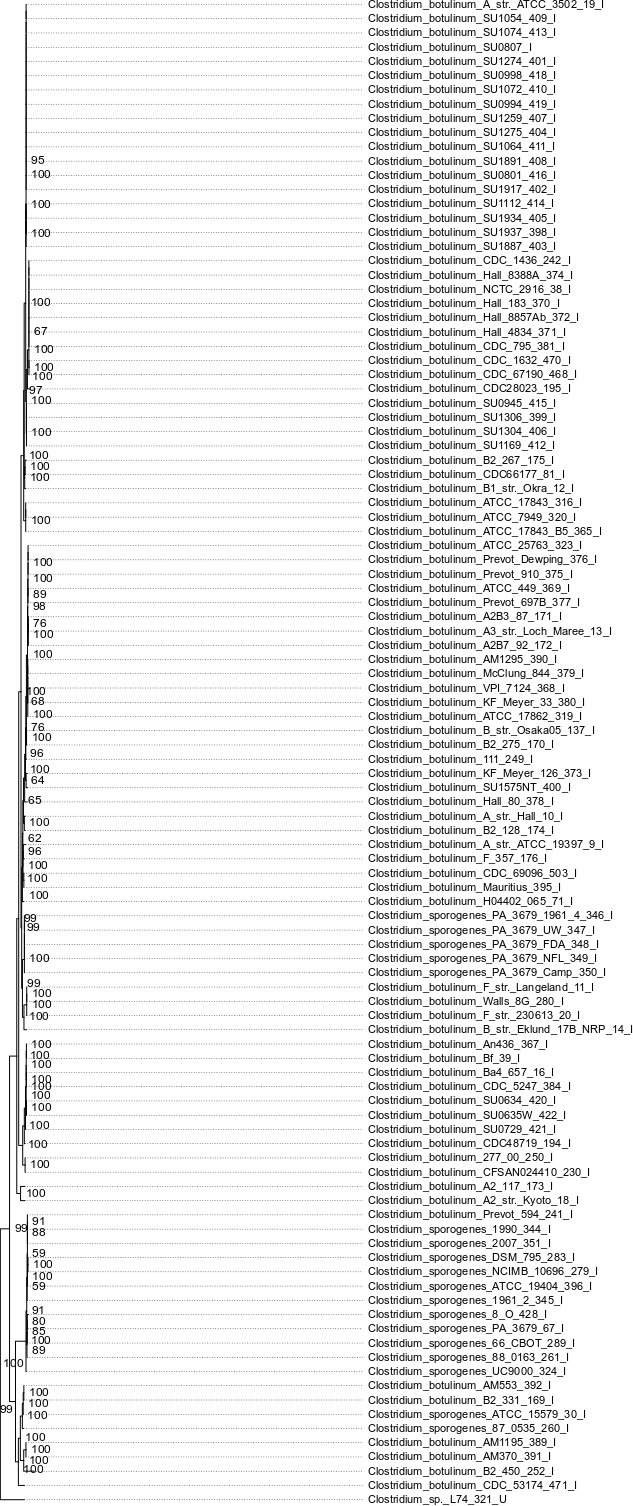
**Figure S1. Zoom-in of subgroup 1 in the species tree shown in Figure 1**

**
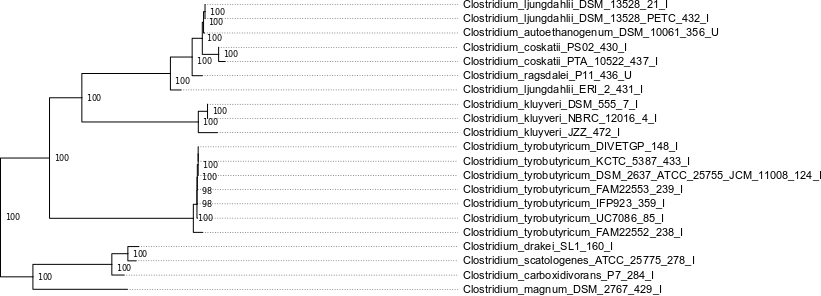
**

**Figure S2. Zoom-in of subgroup 2 in the species tree shown in Figure 1**

**
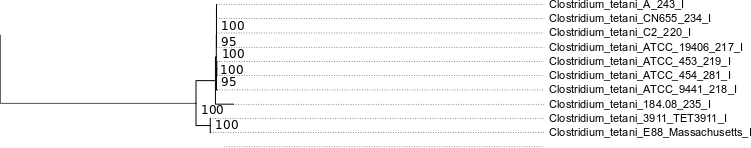
**

**Figure S3. Zoom-in of subgroup 3 in the species tree shown in Figure 1**

**
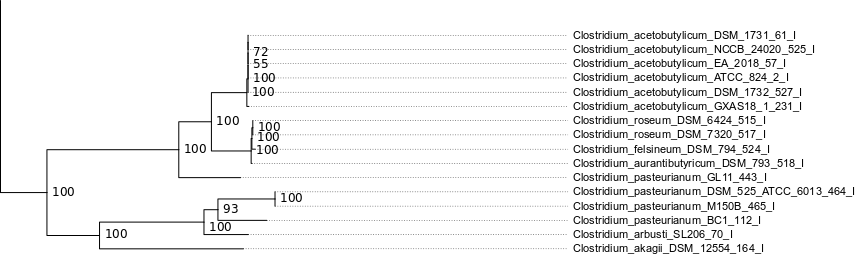
**

**Figure S4. Zoom-in of subgroup 4 in the species tree shown in Figure 1**

**
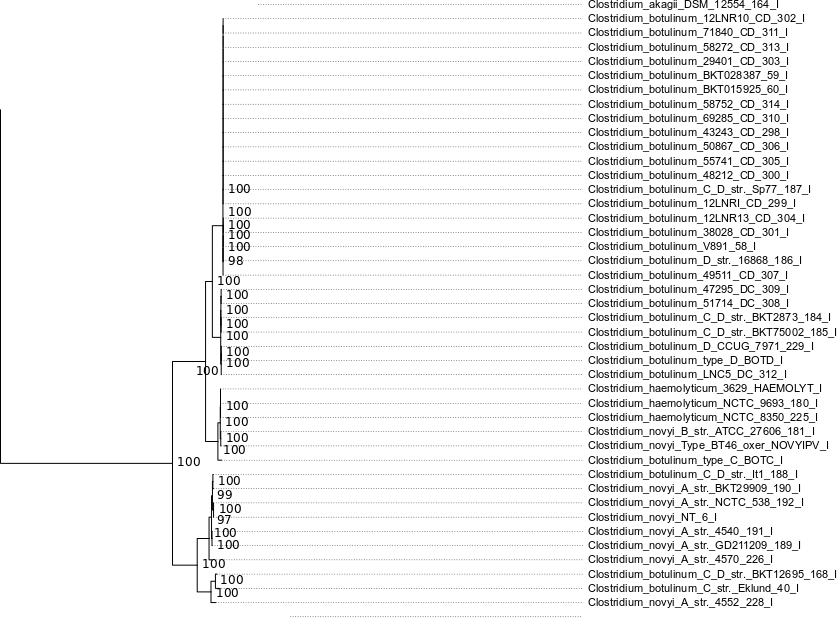
**

**Figure S5. Zoom-in of subgroup 5 in the species tree shown in Figure 1**

**
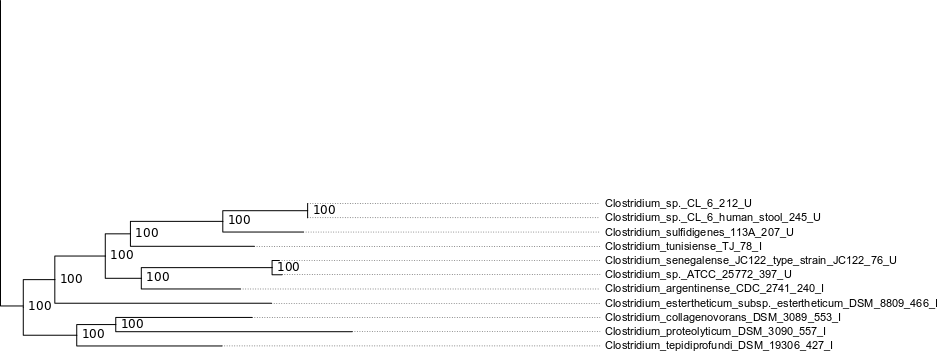
**

**Figure S6. Zoom-in of subgroup 6 in the species tree shown in Figure 1**


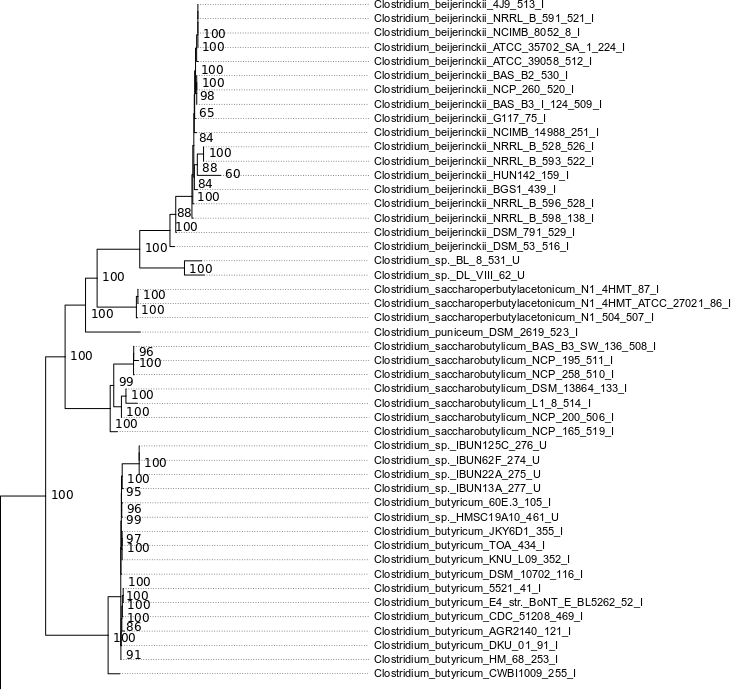
**Figure S7. Zoom-in of subgroup 7 in the species tree shown in Figure 1**

**
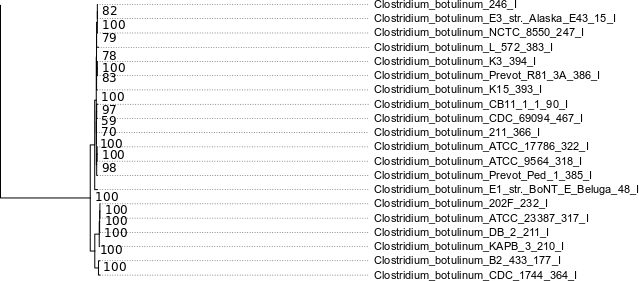
**

**Figure S8. Zoom-in of subgroup 8 in the species tree shown in Figure 1**

**
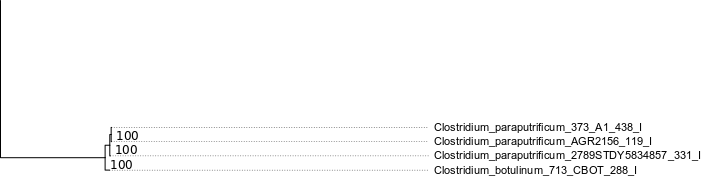
**

**Figure S9. Zoom-in of subgroup 9 in the species tree shown in Figure 1**

**
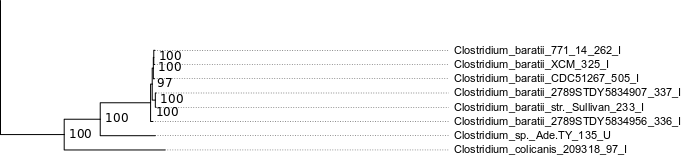
**

**Figure S10. Zoom-in of subgroup 10 in the species tree shown in Figure 1**

**
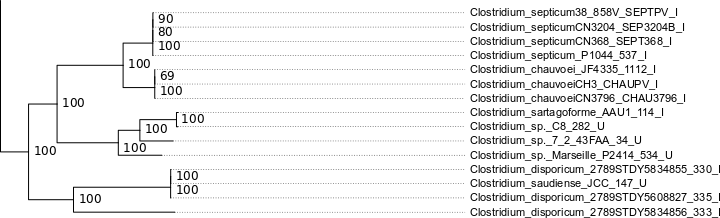
**

**Figure S11. Zoom-in of subgroup 11 in the species tree shown in Figure 1**

**
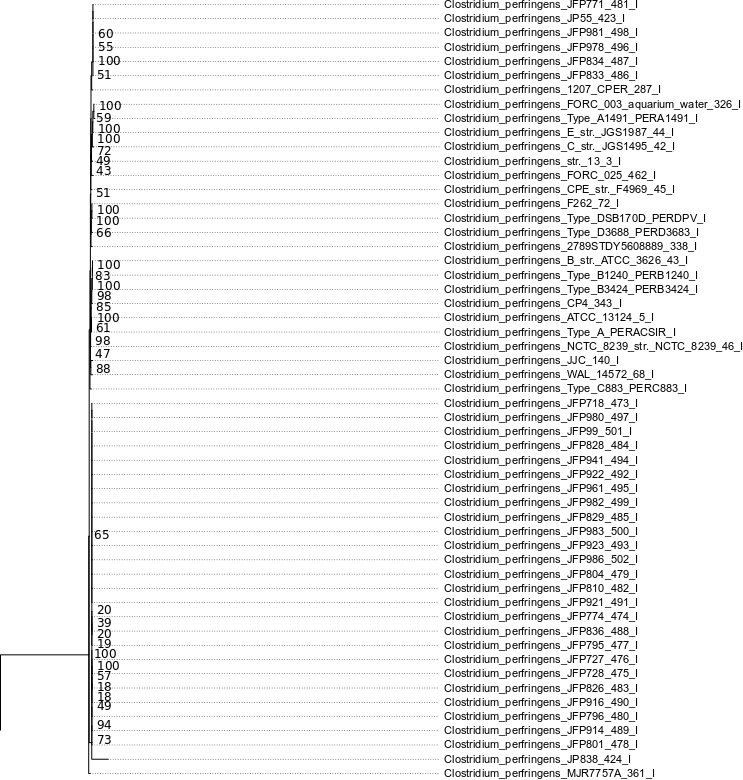
**

**Figure S12. Zoom-in of subgroup 12 in the species tree shown in Figure 1**

**
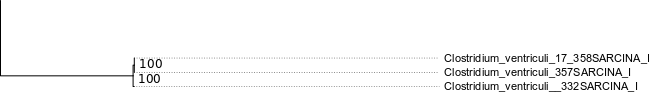
**

**Figure S13. Zoom-in of subgroup 13 in the species tree shown in Figure 1**


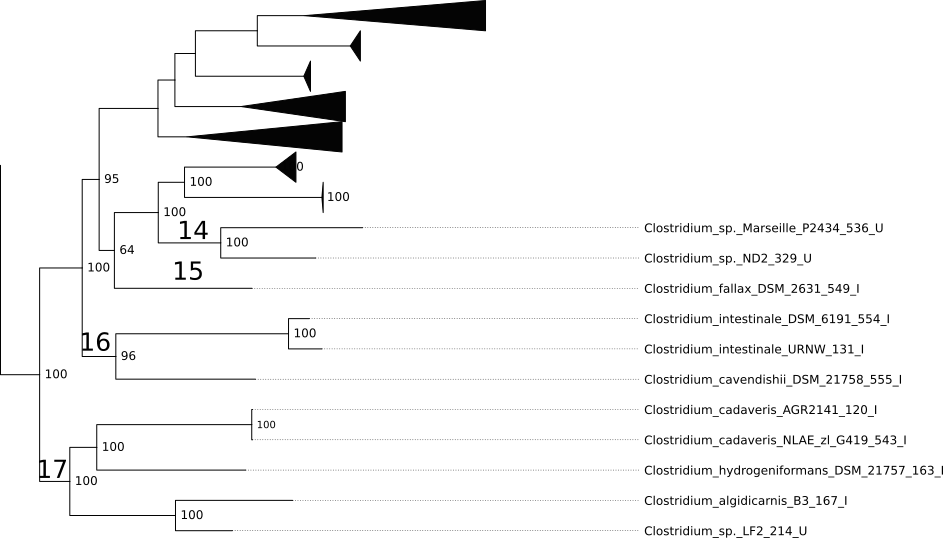
**Figure S14. Zoom-in of subgroups 14-17 in the species tree shown in Figure 1
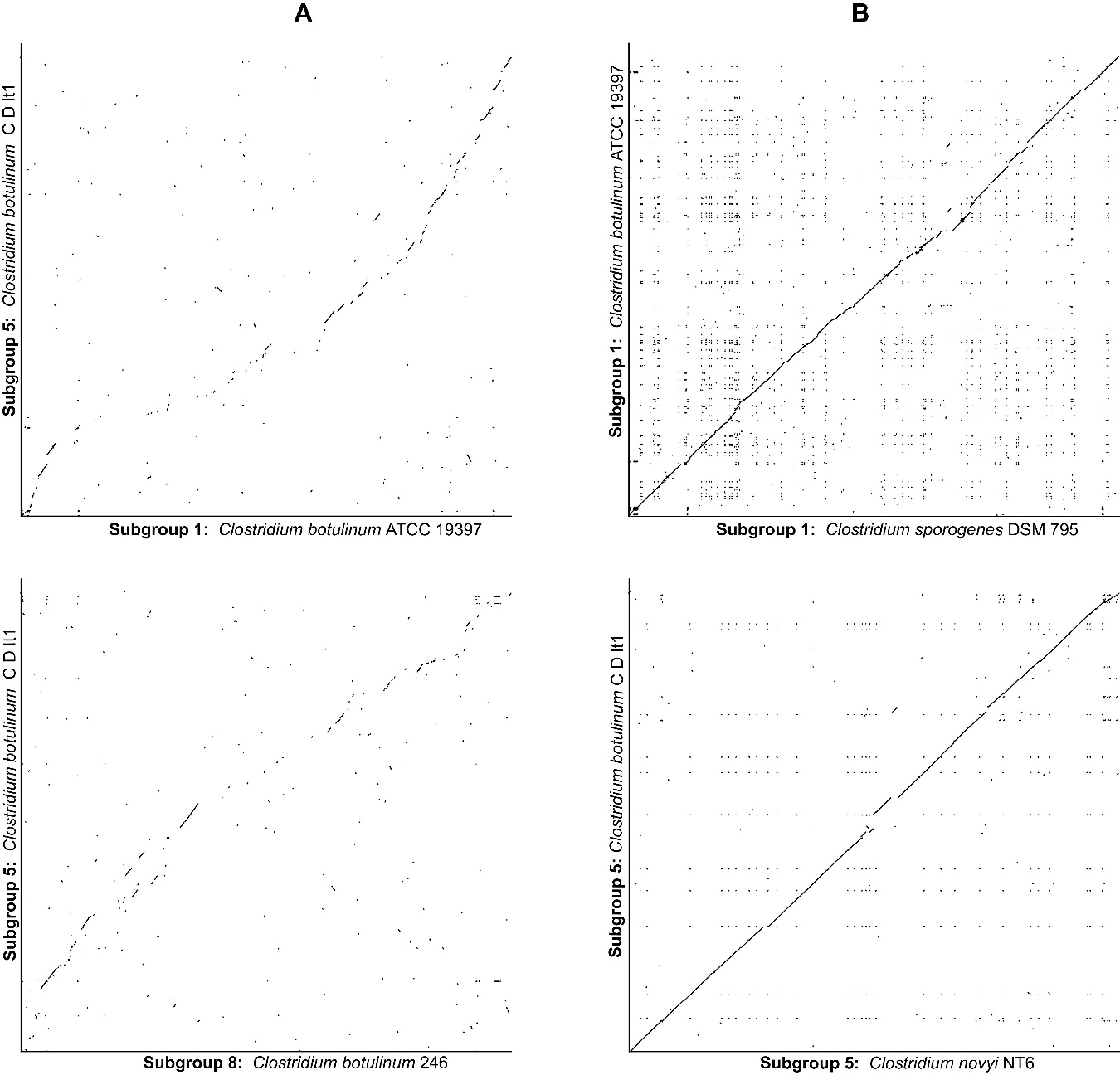
**

**Figure S15. (A)** Synteny among *C. botulinum* strains from different subgroups and **(B)** other closely related strains under distinct species names. DNA sequences of single contig genomes (except for *C. botulinum* C D lt1) were compared using R2CAT (<https://bibiserv2.cebitec.uni-bielefeld.de/cgcat>). Lines represent homologous regions. Syntenic regions are aligned along the diagonal center between the compared genomes. From this representation is clear that *C. botulinum* from Subgroup 1 and *C. sporogenes*; and *C. botulinum* from subgroup 5 and *C. novyi* (defined as a different species) are more syntenic than *C. botulinum* from subgroups 1, 5 and 8 (defined as the same species).


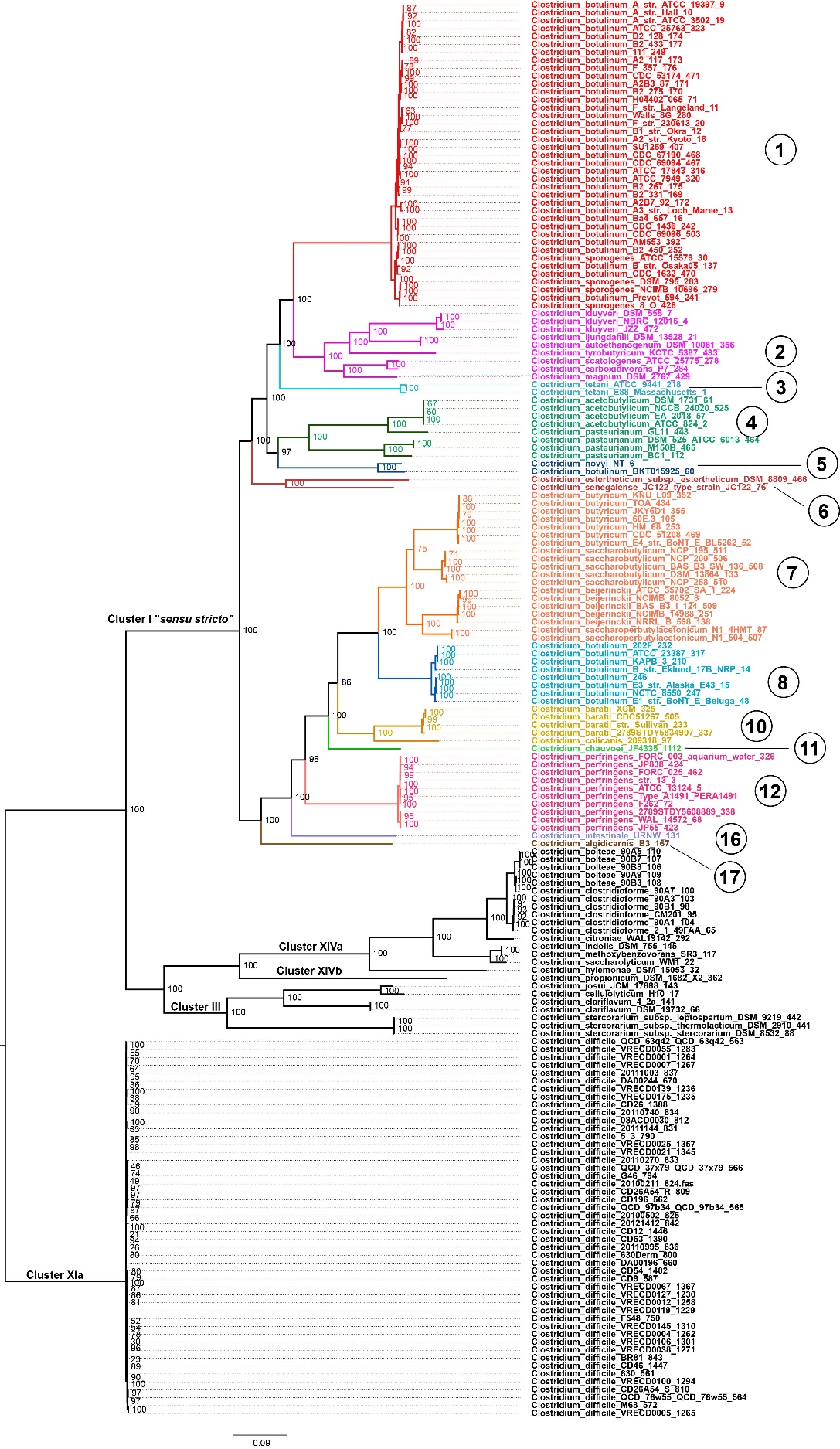


**Figure S16. Second phylogenetic reconstruction of *Clostridium* species.** This phylogeny was constructed using 79 markers conserved across 179 genomes (N50>600,000 and removing unclassified genomes (*Clostridium sp*) from Supplementary Table S1) deposited in the GenBank database and taxonomically defined as *Clostridium*. Each partition (79 proteins) was aligned independently and manually curated. The best independent evolutionary model was determined, and 10,000 bootstrap replicates were performed. The main clades outside and within the *sensu stricto* group (real clostridia) have been defined as taxonomic subgroups (Table 1 in Manuscript). Branch support is shown at each node. Zoom-in of subgroups of Cluster I are shown in Supplementary Figures S17-S29.


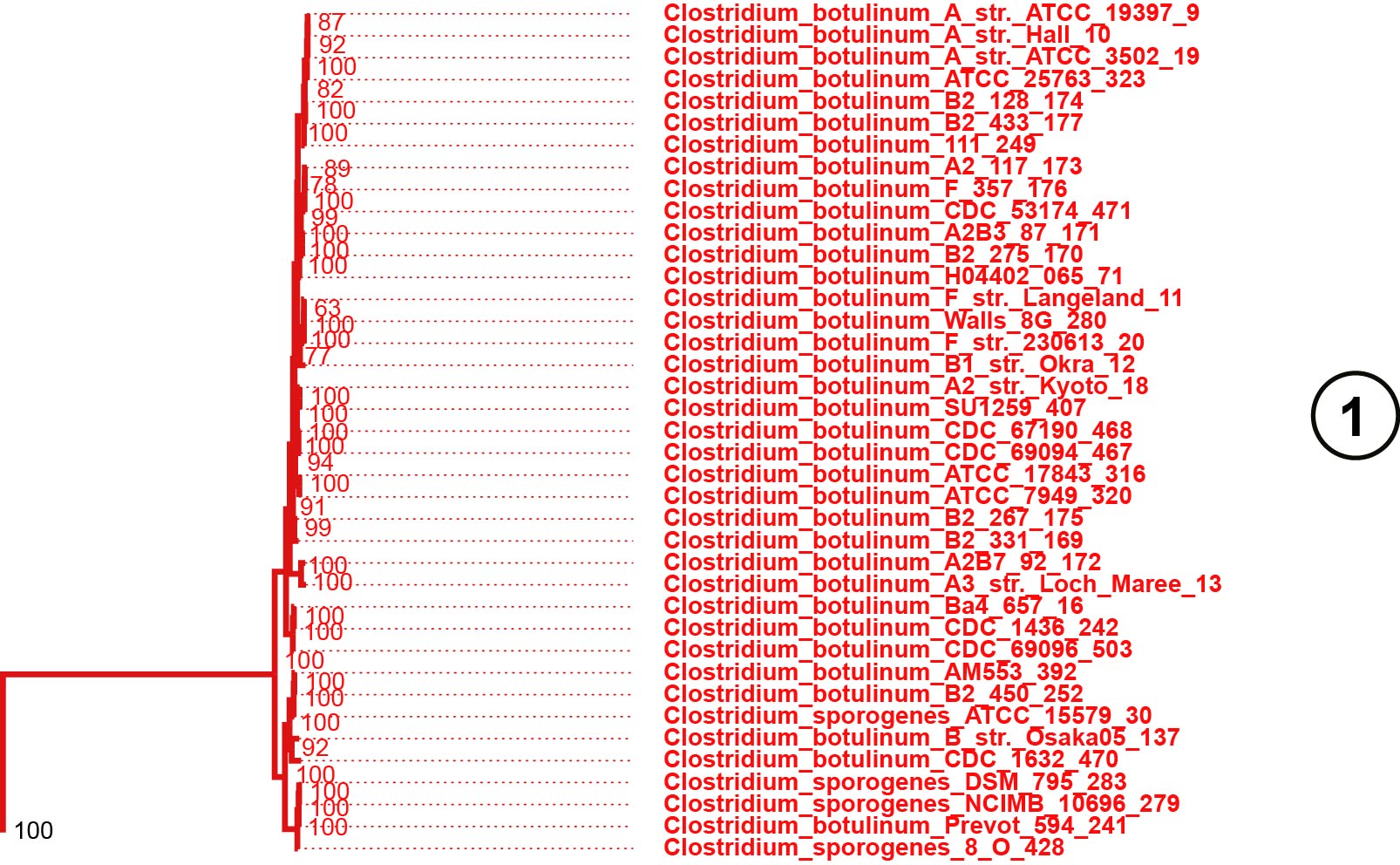


**Figure S17. Zoom-in of subgroup 1 in the species tree shown in Figure S16**


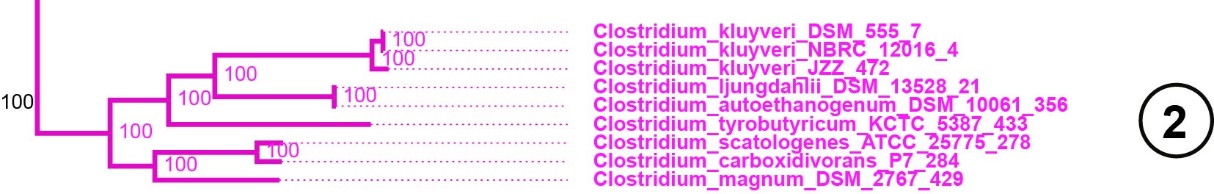


**Figure S18. Zoom-in of subgroup 2 in the species tree shown in Figure S16**


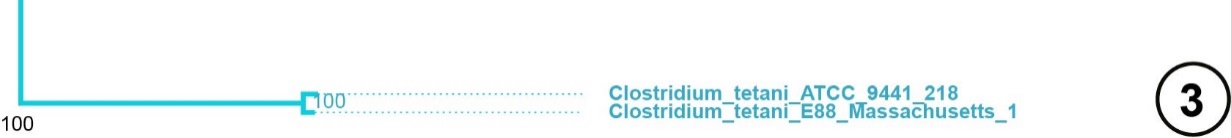


**Figure S19. Zoom-in of subgroup 3 in the species tree shown in Figure S16**


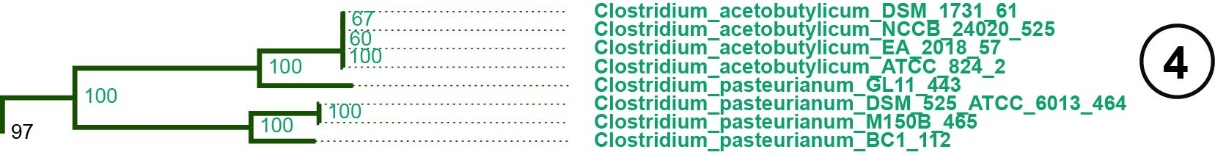


**Figure S20. Zoom-in of subgroup 4 in the species tree shown in Figure S16**


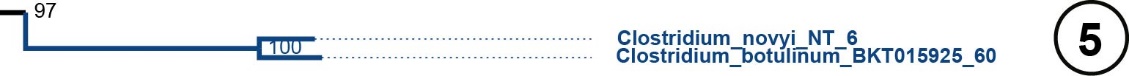


**Figure S21. Zoom-in of subgroup 5 in the species tree shown in Figure S16**


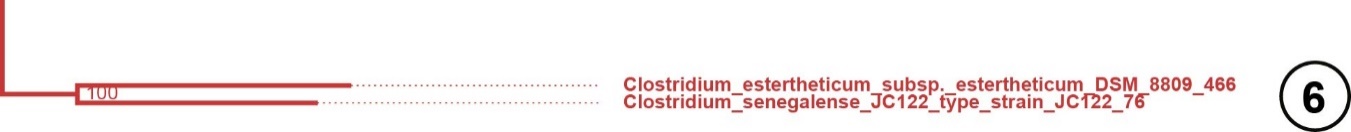


**Figure S22. Zoom-in of subgroup 6 in the species tree shown in Figure S16**


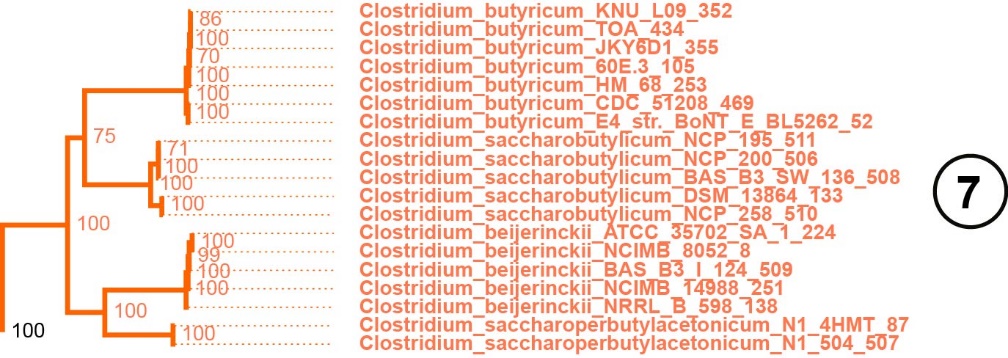


**Figure S23. Zoom-in of subgroup 7 in the species tree shown in Figure S16**


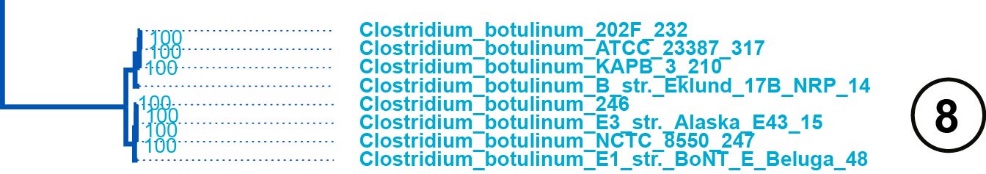


**Figure S24. Zoom-in of subgroup 8 in the species tree shown in Figure S16**


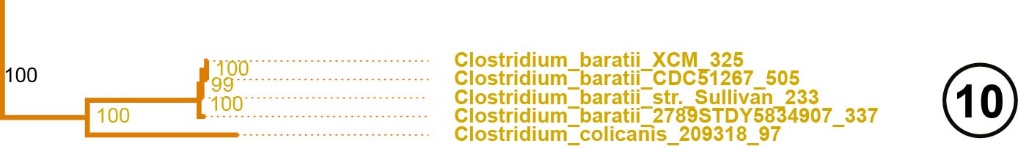


**Figure S25. Zoom-in of subgroup 10 in the species tree shown in Figure S16**


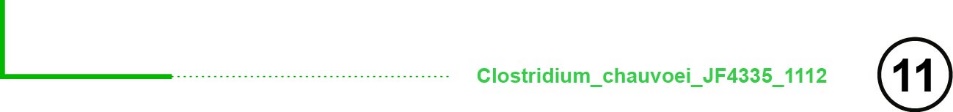


**Figure S26. Zoom-in of subgroup 11 in the species tree shown in Figure S16**


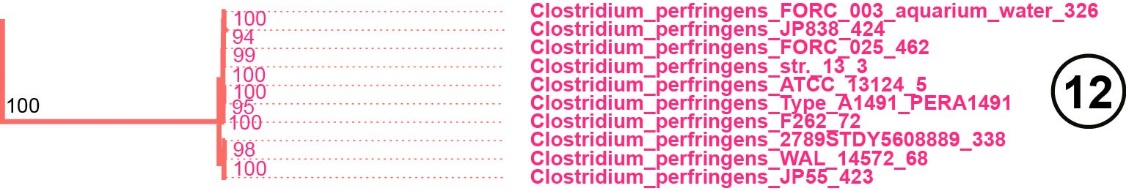


**Figure S27. Zoom-in of subgroup 12 in the species tree shown in Figure S16**


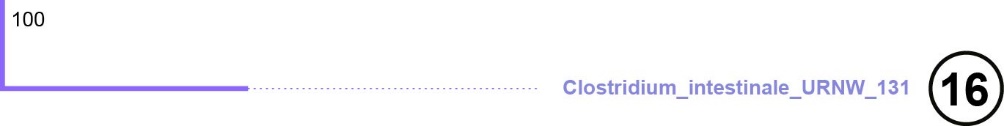


**Figure S28. Zoom-in of subgroup 16 in the species tree shown in Figure S16**


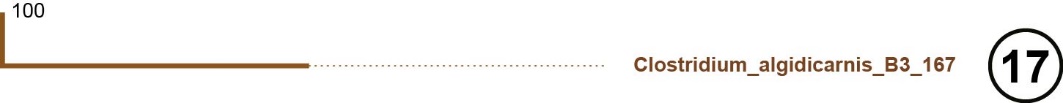


**Figure S29. Zoom-in of subgroup 17 in the species tree shown in Figure S16**


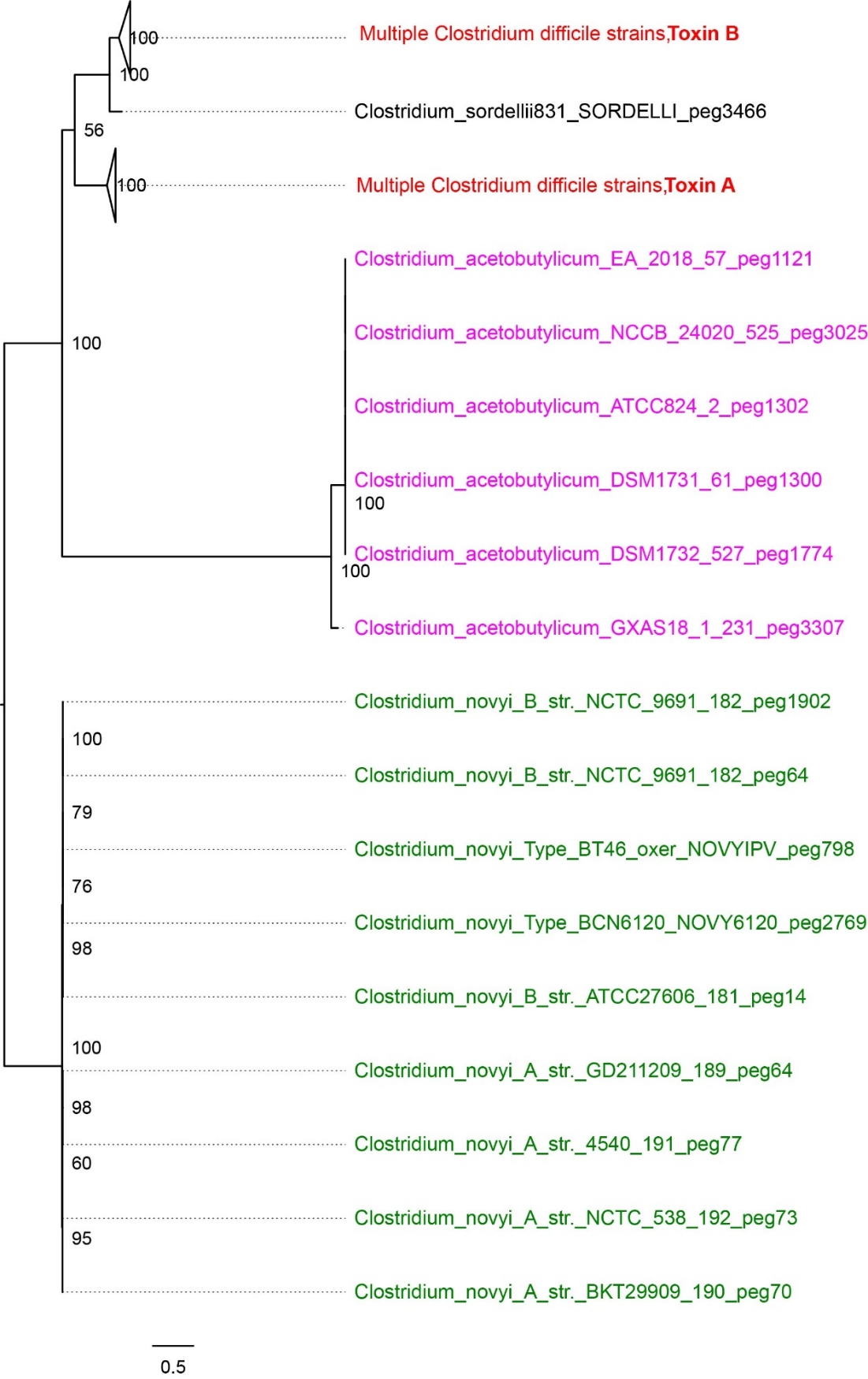


**Figure S30. Phylogenetic reconstruction of *C. difficile* toxins A and B (shown in red) homologous proteins.** Three non-*difficile* strains are included: *C. sordellii*, *C. acetobutylicum* and *C. novyi*.


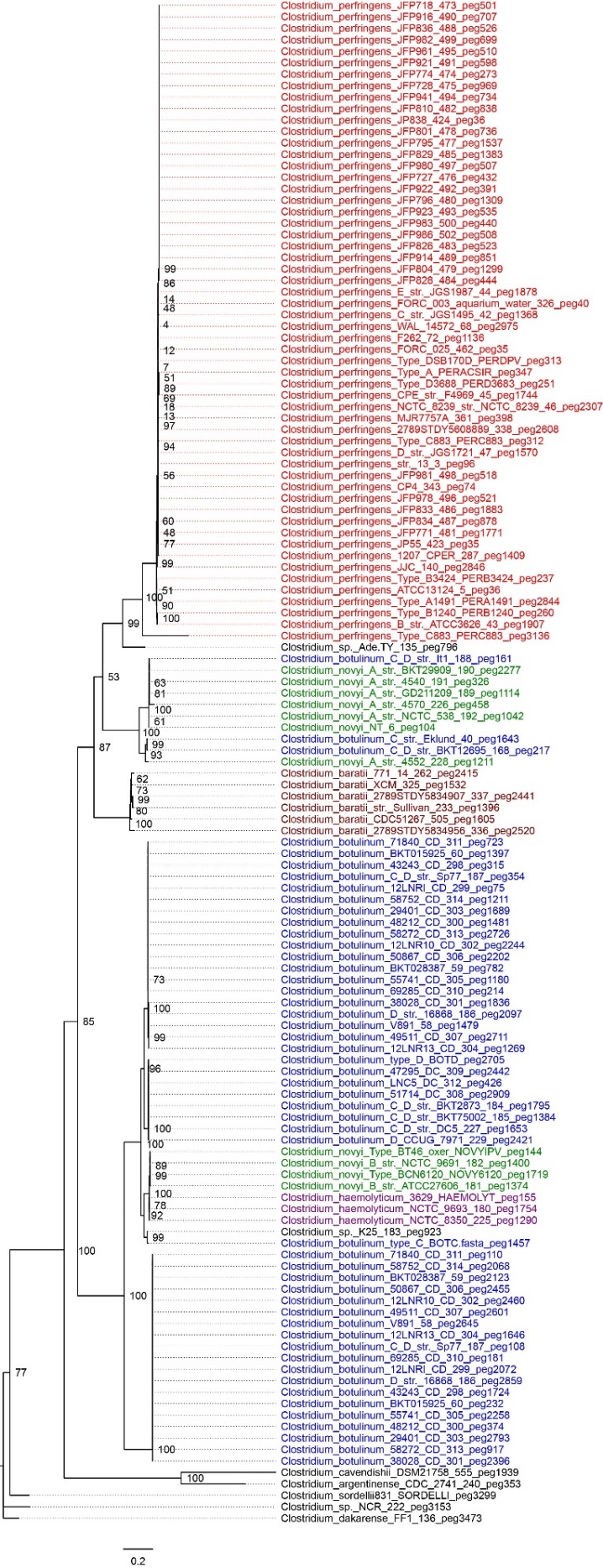


**Figure S31. Phylogenetic reconstruction of *C. perfringens* alpha** **toxin (shown in red) homologous proteins.** Nine non-*perfringens* strains are included: *C. novyi*, *C. botulinum* C and D, *C. baratii*, *C. hemolyticum*, *C. cavendishii*, *C. argentinense*, *C. sordellii* and *C. dakarense*.


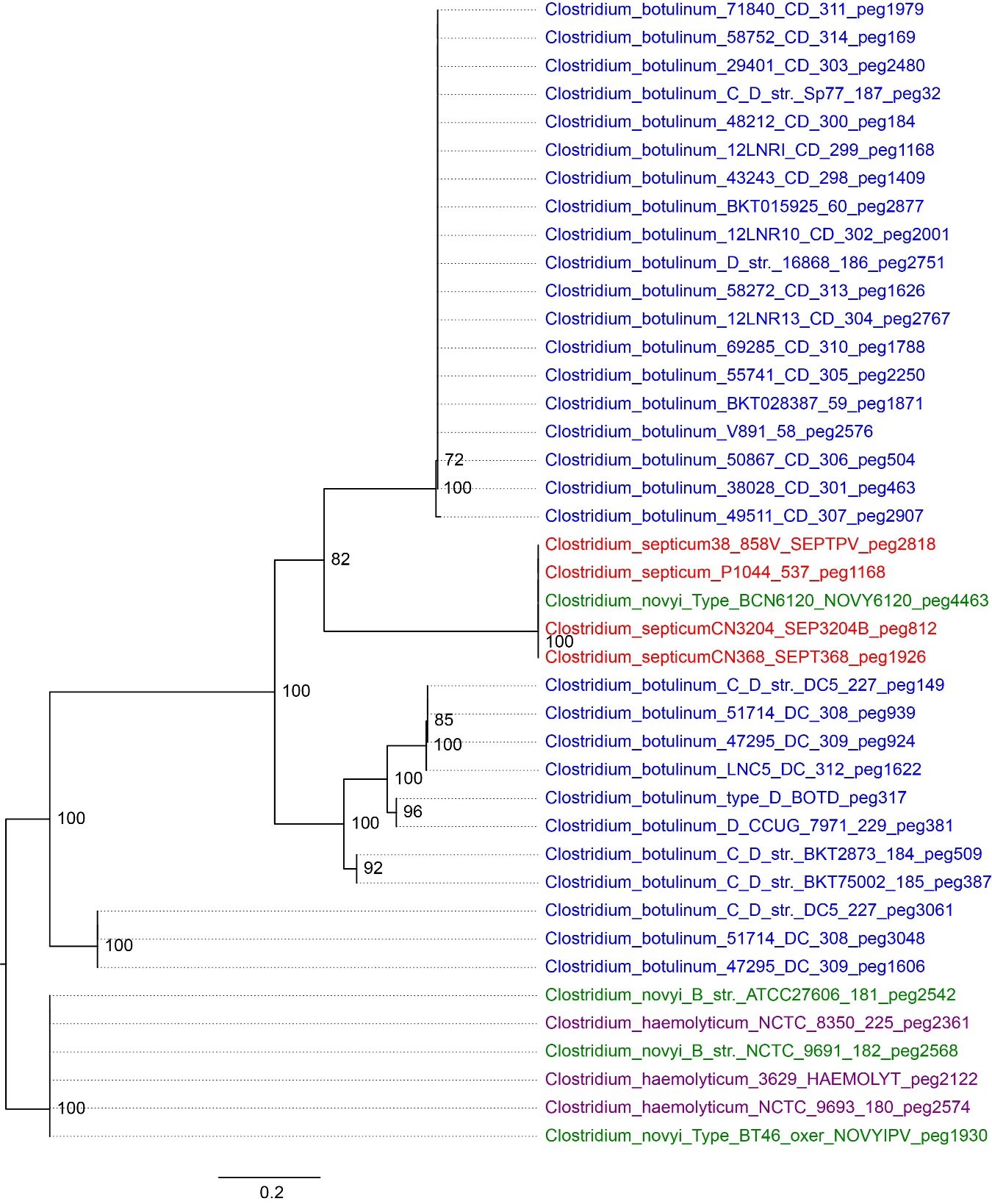


**Figure S32. Phylogenetic reconstruction of *C. septicum* alpha** **toxin (shown in red) homologous proteins.** Four non- *septicum* strains are included: *C. novyi*, *C. haemolyticum* and *C. botulinum* C and D species.

**Figure S33**. **PanGenome analysis of Cluster I strains.** This figure represents the functional content shown as KEGG orthology categories of the core, accessory ad unique genes found in cluster I. Values in the y axis are the percentage of annotated core, accessory or unique functions in each category in the x axis.
